# Natural products and dietary interventions on liver enzymes: an umbrella review and evidence map

**DOI:** 10.3389/fnut.2024.1300860

**Published:** 2024-02-02

**Authors:** Zhongyu Li, Jiao Wu, Yingpan Zhao, Jinjie Song, Yandong Wen

**Affiliations:** ^1^Department of Chinese Medicine, Eye Hospital, China Academy of Chinese Medical Sciences, Beijing, China; ^2^Department of Oncology, Xiyuan Hospital, China Academy of Chinese Medical Sciences, Beijing, China; ^3^Institute of Digestive Diseases, Xiyuan Hospital, China Academy of Chinese Medical Sciences, Beijing, China

**Keywords:** natural products, dietary interventions, liver enzymes, umbrella review, evidence map

## Abstract

**Background:**

The association between natural products and dietary interventions on liver enzymes is unclear; therefore, this study aimed to examine their effects on liver enzymes in adults.

**Methods:**

PubMed, Embase, and Cochrane Library of Systematic Reviews databases were searched from inception until March 2023. The Assessment of Multiple Systematic Reviews-2 (AMSTAR-2) and Grading of Recommendations Assessment, Development, and Evaluation (GRADE) systems were used to assess the methodological and evidence quality, and the therapeutic effects were summarized in a narrative form.

**Results:**

A total of 40 meta-analyses on natural products (*n* = 25), dietary supplements (*n* = 10), and dietary patterns (*n* = 5) were evaluated, and results were presented in a narrative form. The overall methodological quality of the included studies was relatively poor. The results indicated that positive effects were observed for nigella sativa, garlic, artichoke, curcumin, silymarin, vitamin E, vitamin D, L-carnitine, propolis, and polyunsaturated fatty acids on certain liver enzymes. The dietary patterns, including high-protein, Mediterranean, and calorie-restriction diets and evening snacks, may reduce liver enzymes; however, other supplements and herbs did not reduce liver enzyme levels or have minimal effects. The evidence quality was generally weak given the risk of bias, heterogeneity, and imprecision.

**Conclusion:**

This umbrella review suggests that natural products and dietary interventions have beneficial therapeutic effects on liver enzymes levels. Further clinical trials are necessary to establish the effectiveness of supplements that reduce liver enzymes.

## 1 Introduction

The liver is an important metabolic organ and is rich in enzyme systems that play an important role in the metabolism and protein synthesis in the body (1). Specifically, it is applied to degrade toxins, secrete bile, store glycogen, and metabolize drugs (2). These physiological functions can be disrupted by liver disease and/or drug use (3, 4). Several serum liver enzymes, including alanine aminotransferase (ALT), aspartate aminotransferase (AST), gamma-glutamine transferase (GGT), and alkaline phosphatase (ALP), are highly sensitive to liver dysfunction and injury (5, 6). When hepatocytes are damaged enzymes within the cytoplasm are released, raising serum enzyme activity, which can reflect various pathological conditions in the liver. Although chronic liver injury patients are usually asymptomatic, the liver enzymes appear generally significantly higher (7). In recent decades, several studies have indicated that liver enzyme disorders are associated with various diseases, such as chronic obstructive pulmonary disease (8), metabolic diseases (9), cardiovascular disease (10), and type 2 diabetes mellitus (T2DM) (11). Meanwhile, liver enzyme disorders are a significant predictive factors for increased patient mortality (12). Hence, there is an urgent clinical need to improve liver dysfunction.

The application of complementary and alternative therapies has increased in recent years, as nearly half of the US population is reported using at least one dietary supplement, and 10% of the participants used at least four dietary supplements (13–15). Similarly, the use of herbal medicines has also increased in several Asian countries (16). Another study reported similar percentages of herbs used as complementary and alternative medicines to treat chronic liver disease (17, 18). The herbal and dietary supplements have been found to promote liver function (19, 20). For example, silymarin acts as a free radical scavenger and has been reported to modulate elevated liver enzymes (21). Similarly, resveratrol reduces diet-induced liver fat accumulation via increased fatty acid oxidation and lipogenesis reduction, exerting hepatoprotective effects in liver injury, and thus, could be used to develop hepatoprotective drugs (20, 22). Natural products also offer a significant supply of antioxidants that can regulate liver enzymes, are affordable, have fewer side effects, and are more easily accessible than their synthetic counterparts (23, 24).

In recent decades, many observational studies and randomized controlled trials (RCTs) focusing on the association between supplement intake and liver enzymes levels have been published (25, 26). Thus, before developing consensus and guideline policies, a comprehensive assessment of the quality of available evidence on the association between natural products and dietary supplements and liver injury outcomes is required. It is imperative to conduct an integrated review of various herbs and dietary interventions since not all are reported to have beneficial effects on liver function (4). Therefore, to summarize the evidence of the effect of natural products and dietary interventions on liver enzymes, a comprehensive review of meta-analyses was conducted to help elucidate their effects in regulating liver enzyme status.

## 2 Materials and methods

Umbrella reviews are systematic searches, integrating and evaluating available evidence on specific exposure factors and health outcomes in systematic reviews and/or meta-analyses (27). The results of natural products, dietary supplements, and dietary patterns on liver enzymes in the meta-analysis were reviewed, excluding systematic reviews without meta-analysis.

### 2.1 Literature search

PubMed, Embase and Cochrane Library of Systematic Reviews databases were searched from their inception to March 2023 to identify meta-analyses of natural products, dietary supplements, and dietary patterns on liver enzymes. We used the following search terms: (alanine transaminase or aspartate aminotransferases or gamma-glutamyl transferase or ALS or AST or GGT or ALP or liver enzymes or liver function) and (systematic review or meta-analysis) (see Supplementary Table 1). Two reviewers (J.W. and J.J.S.) independently screened titles and/or abstracts, and selected potential articles for full-text review. Then, they independently reviewed the full article for eligibility. In case of any disagreement, a third reviewer (Y.D.W) was engaged to resolve the issue. Moreover, references for eligible articles were manually searched to identify additional studies that fulfilled the inclusion criteria.

### 2.2 Inclusion and exclusion criteria

All articles that were meta-analyses and conducted using systematic reviews were deemed eligible for the study. The specific criteria for inclusion regarding population, interventions or exposures, comparators, outcomes, and study design were as follows: (1) Participants: adult subjects with or without any health condition; (2) Interventions: oral natural products, dietary supplements as well as dietary patterns; (3) Controls: placebo, no treatment, and conventional treatments; (4) Outcomes: serum liver enzymes, including ATL, AST, GGT, and ALP levels; (5) Study type: meta-analysis based on RCTs. Non-human studies, genetics studies, original studies, and conference abstracts were excluded. Similarly, meta-analyses with fewer than two primary studies and studies on combinations of multiple herbs were also excluded. The most recent, most significant, and updated meta-analyses were preferred if multiple meta-analyses were performed on the same interventions and outcomes (28). When the most recent meta-analysis was not the largest in number, one that included more primary studies was selected.

### 2.3 Data extraction

The data from eligible articles were extracted by two independent reviewers (J.W. and Y.P.Z.). The extracted data included the first author, publication year, study population, number of RCTs, sample size, supplements (herbs and dietary) and dietary patterns, dosage, frequency of administration, form of intervention, treatment duration, registration information, risk of bias tool, and outcomes of interest. Any discrepancies in the data extraction were resolved by consensus.

### 2.4 Assessing the methodological quality and quality of evidence

The methodological quality of the meta-analysis was evaluated by the Assessment of Multiple Systematic Reviews (AMSTAR)-2 checklist, which is a validated and reliable tool for appraising systematic reviews and interventional and observational meta-analyses (29). AMSTAR-2 checklist contains an assessment on search, analysis, and transparency of meta-analysis, and classifies studies as “high,” “moderate,” “low,” and “critically low” quality.

Similarly, the Grades of Recommendations, Assessment, Development, and Evaluations (GRADE) system was used to assess the quality of evidence for outcomes (30), which provides clear criteria for grading the quality of the evidence, including bias risk, imprecision, inconsistency, indirectness, and publication bias. An evidence map was developed to present the plausible benefits of each intervention and the certainty of evidence. Based on the GRADE system (GRADEpro GDT),^1^ the certainty of the evidence was categorized into “high,” “moderate,” “low,” or “very low” quality.

### 2.5 Statistical analysis

The results were generated using a narrative approach (31), including the risk ratio (RR), odds ratio (OR), mean difference (MD), weighted mean difference (WMD), standardized mean difference (SMD), 95% confidence interval (CI), and *p*-value. The heterogeneity of each meta-analysis is presented by I^2^ statistics, and the cutoff values for “low,” “moderate,” and “high” heterogeneity were 25, 50, and 75%, respectively. If the included meta-analyses present subgroup analyses were conducted based on the factors such as sex, age, health status, country, dose, and study duration, they will be reported. In addition, the publication bias was also investigated using funnel plots, Begg’s or Egger’s regressions. Data were analyzed using Excel 2016 (Microsoft Corporation, WA, USA).

## 3 Results

### 3.1 Literature screening results

**FIGURE 1:**
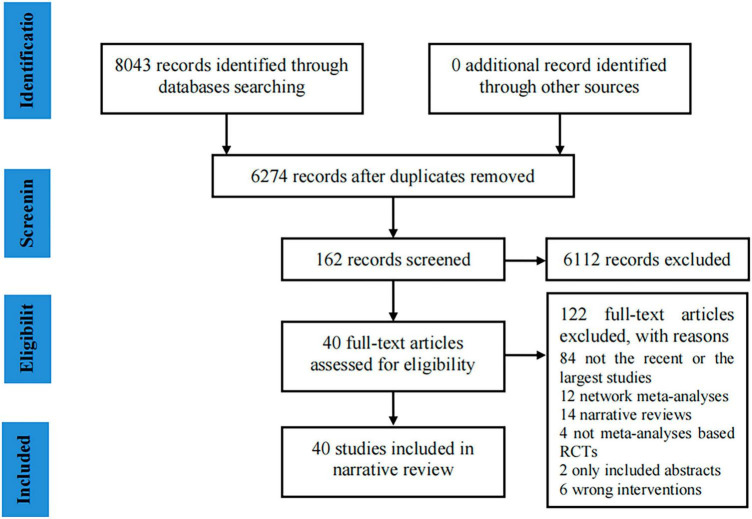
Flow chart for the included studies.

The preliminary search identified 8,043 potential articles, and after removing duplicates, 6,274 records remained. Subsequently, 6,112 articles were excluded after screening the titles and abstracts. The full texts of the 162 selected records were further evaluated, and the final 40 articles (32–71) were included for analysis, as revealed in Figure 1, displaying the study selection flowchart.

### 3.2 Basic characteristics

The basic characteristics of included meta-analysis are presented in Table 1, where all published meta-analyses in peer-reviewed journals between 2013 and 2023 are given. The included meta-analyses were distributed across ten geographic regions, including 20 from Iran, ten from China, two from Greece and Korea, and one from Pakistan, Malaysia, Canada, the United States, the United Kingdom, and Egypt. The number of RCTs included in the meta-analysis ranged from 2 to 26, with subjects between 150 and 3,637, respectively. Twenty-five meta-analyses were found to have used only placebo controls, with the remaining studies using placebo, no treatment, or conventional regimens as controlled treatments. Moreover, seven meta-analyses reported dosing frequencies that ranged from two to three times daily. The treatment period ranged from 2 weeks to 5 years. Sixteen meta-analyses registered their protocols on public platforms, whereas 24 lacked statements. For the risk bias assessment, 35 meta-analyses used the Cochrane risk of bias, three used the Jadad scale, and two were not evaluated in this domain.

**TABLE 1:** The basic characteristics of included studies.

References	Country	Health status	Interventions/ comparations	Number of primary studies	Sample size	Dose	Frequ-ency	Form	Duration	Registration information	Bias of risk assess-ment	Reported outcomes	Safety
(51)	Iran	Adults (T2DM = 3, RA = 1, Prediabetes = 1, NAFLD = 1, Impaired glucose tolerance = 1)	Cinnamon/Placebo	7	256	120–10,000 mg/d	Twice = 1, Third = 2 NR = 4	Water soluble extract = 1, capsules = 6	4–13 weeks	OSF (ID: https://doi.org/10.17605/OSF.IO/XSNYW)	Cochrane	AST, ALT, ALP	NR
(71)	Iran	Adults (Healthy individuals = 5, obese patients with metabolic disorders = 20, NAFLD and liver diseases = 8, heart diseases = 2, chronic obstructive pulmonary disease = 1, osteoarthritis = 1)	Resveratrol/Placebo	37	2,114	8–3,000 mg/d	NR	NR	4–48 weeks	PROSPERO (CRD4202232 6132)	Cochrane	AST, ALT, ALP, GGT	NR
(70)	Canada	Adults (NIDDM = 1, T1DM/T2DM = 2, Healthy = 4, hypertension = 2, hyperlipidemia = 1)	Non-nutritive sweetener/Placebo	10	854	30–1,800 mg/d	NR	NR	4 weeks to 104 weeks	PROSPERO (CRD4202125 0067)	Cochrane	AST, ALT, GGT	NR
(58)	Pakistan	Adults (Mets = 1, T2DM = 1, hypertension = 1, cardiovascular disease = 2, healthy adults = 1, overweight/obese = 2)	Grape products/Placebo	8	291	200–60 g/d	NR	Powder = 3, extract = 5	3 weeks to 12 months	No	Cochrane	AST, ALT	NR
(64)	Malaysia	NAFLD	Curcumin/Placebo	16	1,028	50–1,500 mg/d	NR	Capsules = 16	8–24 weeks	PROSPERO (CRD4202122 9160)	Cochrane	ALT, AST	Yes
	Iran	Adults	Almond/Placebo, no intervention	26	1,750	5 and 85 g/d	NR	NR	4 and 20 weeks	No	Cochrane	ALT, AST, GGT	NR
	China	Metabolic disorders (T2DM = 4, NAFLD = 3, dyslipidemia = 1, hyperlipidemia = 3, pre-diabetes or early untreated diabetes = 1)	Anthocyanins/ Placebo	12	893	0.77–600 mg/d	NR	Extract = 7, purified anthocyanins = 3, Juice = 1, blueberry = 1	4–12 weeks	No	Cochrane	AST, ALT	NR
(57)	Iran	Adults	Betaine/Control	12	528	1.5–20 g/day	NR	NR	2–52 weeks	No	Cochrane	AST, ALT, GGT	NR
(66)	Iran	NAFLD	Garlic/Placebo	2	186	800 mg to 1.6 g/d	NR	Powder = 2	12–15 weeks	Tabriz University of Medical Sciences (ID 38774)	Cochrane	AST, ALT	No
(62)	China	Individuals with any health condition (Fatty liver disease = 1, T2DM = 1, postmenopausal women = 1, Breast cancer = 1)	Tocotrienols/Placebo	4	437	400 mg to 860 mg/d	Twice = 3, NR = 1	NR	8 weeks to 5 years	No	Cochrane	AST, ALT	NR
(59)	Iran	Adults (Obese or overweight = 13, healthy adults = 4, postmenopausal women with T2DM = 1, NAFLD = 1)	Conjugated linoleic acid/Placebo, control diet	19	848	1.17–10.8 g/d	NR	NR	3–104 weeks	PROSPERO (CRD4202233 1111)	Cochrane	AST, ALT	NR
(65)	Korea	Chronic Liver Disease (Chronic hepatitis = 6, NASH = 1, NAFLD = 2, NAFLD with T2DM = 2, hepatic Encephalopathy = 3)	L-Carnitine/Placebo, no L-Carnitine	14	1,217	600 mg to 4 g/d	NR	NR	NR	PROSPERO (CRD4202233 2856)	Cochrane	AST, ALT	NR
(1)	USA	Adults	High-protein diet/standard protein diet, conventional diet	5	558	NR	NR	NR	12 weeks to 12 months	PROSPERO (CRD4201914 0511)	Cochrane	AST, ALT	Yes
(67)	Iran	Adults (NAFLD = 6, obstructive sleep apnea = 1, obese patients with diabetes = 1, subjects with high cardiovascular Risk = 1, CVD = 1)	Mediterranean diet/low-fat diet, energy restriction diet, American Diabetes Association diet and without any dietary treatment	10	705	NR	NR	NR	1.5–72 months	PROSPERO (CRD4202123 3214)	Cochrane	AST, ALT, GGT	NR
(60)	UK	NAFLD	Mediterranean diet and calorie restriction/Standard care	21	3,037	NR	NR	NR	2–104 weeks	PROSPERO (CRD4201911 8537)	Cochrane	AST, ALT	NR
(61)	Egypt	Fatty Liver	Artichoke/Control	5	333	150–2,700 mg/d	NR	Tablet = 3; powder extract = 2	4–24 weeks	PROSPERO (CRD4202018 2502)	Cochrane	AST, ALT, ALP	NR
(45)	Iran	Adults (hypertension = 2, Obese = 1, xerotic skin = 1, NAFLD = 1, hypercholesterolemic = 1, health = 2, Mets = 1)	Green coffee bean extract/Placebo	8	NR	50–1,200 mg/d	NR	NR	8–12 weeks	No	Cochrane	ALT, AST, ALP, GTP	NR
(54)	China	NAFLD	Nigella sativa/Placebo	5	358	75 mg to 2 g/d	Twice = 2, tid = 1, per = 2	Powder = 3; oil = 2	8–24 weeks	No	Cochrane	AST, ALT	Yes
(48)	Iran	NR	Propolis/Placebo	5	313	500 mg to 1,000 mg	NR	NR	8 week to 24 months	No	NR	AST, ALT	NR
(47)	Iran	Mets and related disorders (Hyperlipidemia = 1, NAFLD = 1)	Sumac fruit/Placebo	2	150	1,000 to 2,000 mg/d	NR	NR	6–12 weeks	PROSPERO (CRD4202122 7425)	Cochrane	AST, ALT	NR
(53)	Iran	NAFLD	Vitamin D/Placebo	16	657	NR	NR	NR	4–48 weeks	No	Cochrane	AST, ALT, ALP, GGT	NR
(55)	Greece	Adults (NAFLD = 4, NASH = 3)	Vitamin E/Placebo	7	465	400 to 800 mg/d	Twice = 2, NR = 5	NR	8–96 weeks	OSF (https://osf.io/w4fh3/)	Cochrane	AST, ALT	NR
(49)	Greece	NAFLD	Silymarin/Placebo	8	622	140 to 2,100 mg/d	NR	NR	8–48 months	OSF (https://osf.io/2b54z/)	Cochrane	AST, ALT	NR
(52)	Iran	NAFLD	Resveratrol/Placebo	5	216	300 to 1,500 mg/d	NR	NR	12–24 weeks	No	Cochrane	AST, ALT, ALP, GGT	NR
(50)	Iran	Adults (Schizophrenia = 1, depressive disorder = 1, diabetes = 4, NAFLD = 2, and healthy populations = 2)	Saffron/Placebo	12	608	30 to 100 mg/d	NR	Tablets = 5, capsule = 6, powder = 1	4–12 weeks	No	Cochrane	ALT, AST, ALP	NR
(46)	Iran	T2DM	Chromium/placebo	3	160	400 to 1,000 μg/d	NR	NR	12–25 weeks	No	Cochrane	ALT, AST	NR
(56)	China	NAFLD	Soy diet/control	3	172		NR	NR	8 weeks	No	Jadad Scores	AST, ALT	NR
(40)	Iran	Adults (T2DM = 4, healthy subjects = 4, NAFLD = 1, postmenopausal women = 1, ovarian cancer = 1, Mets = 1, overweight and obese adults = 1, ischemic stroke = 1)	Ginseng/Placebo	14	992	0.75–6 g/day	NR	NR	2–24 weeks	No	Cochrane	ALT, AST, GGT, ALP	NR
(39)	Iran	NR (Allograft renal transplant operation = 1, Low cardiovascular risk = 1, NAFLD = 1, T2DM = 1)	Berberine/Placebo	4	430	600 to 1,500 mg/d	NR	NR	1 to 4 months	No	Cochrane	AST, ALT	NR
(44)	Iran	Adults (Stable angina = 1; hypertension = 2; kidney disease = 2; T2DM = 2; Obese = 3; Liver diseases = 2; healthy individuals = 4; osteoporosis = 1; hypercholesterolemia = 1; Menopause women = 1)	Nigella sativa/Placebo	19	1,295	500 mg to 6,000 mg/d	NR	Oil = 12; powder = 7	28 to 294 days	PROSPERO (CRD42018102 229)	Cochrane	ALT, AST, ALP	NR
(42)	Iran	NR (Adults with elevated serum Gamma-Glutamyl Transferase = 1, coronary artery diseases = 1, mild hypercholesterolemia = 1, uncontrolled hypertension = 1, peritoneal dialysis patient = 1, arsenical palmer keratosis = 1)	Garlic/Placebo	6	301	10 mg to 2.4 g/d;4 ml/d	NR	Capsule = 1, powder = 1, tablet = 2, extract = 2	8 weeks to 1 year	No	Cochrane	AST, ALT	NR
(41)	Iran	Adults (NASH = 1, NAFLD = 4, T2DM = 3, healthy subjects = 2, obese and overweight = 4, postmenopausal women = 2, hypercholesterolemia = 1)	Green tea/Placebo	17	NR	300 mg to 1,500 mg/d	NR	NR	3 to 48 weeks	No	Cochrane	AST, ALT, ALP	NR
(43)	Iran	Adults (Thyroid patients = 2, T2DM with NAFLD = 1, NAFLD = 1, cystic acne = 1, NASH = 4, patients with suspected acute myocardial infarction = 1, cirrhotic = 1, Healthy = 3, hepatocellular carcinoma = 1, hemodialysis = 2, obese = 1)	L-carnitine/Placebo, no treatment	18	1,161	500 to 4,000 mg/d	Twice = 1, NR = 17	NR	2 to 48 weeks	Shahid Sadoughi University of Medical Sciences (IR.SSU.SPH. REC.1398.016).	Cochrane	AST, ALT, ALP, GGT	NR
(38)	China	Antituberculosis Drug-Induced Liver Injury	Silymarin/Placebo	3	747	210 to 420 mg/d	Tid = 1, bid = 2	NR	2 to 8 weeks	No	Cochrane	AST, ALT, ALP	Yes
(36)	South Korea	NAFLD	Low carbohydrate diet/low fat diet group	5	165	NR	NR	NR	2 weeks to 6 months	No	NR	AST, ALT	NR
(37)	China, Taiwan	Liver cirrhosis	Late evening snacks/non-late evening snacks	6	241	200 to 320 kcal.	09:00 PM and 10:00 PM	NR	2 weeks to 12 months	No	Cochrane	AST, ALT	NR
(34)	China	NAFLD	Omega-3 Fatty Acid/Placebo, no treatment	8	530	830 to 9,000 mg/d	NR	Capsule = 8	8 weeks to 18 months	No	Jadad Scores	ALT, AST, GGT	NR
(35)	China	NAFLD	Berberine/lifestyle intervention, other medicines	5	467	0.9–1.5 g/d	Tid = 5	NR	12 to 16 weeks	No	Cochrane	AST, ALT	NR
(33)	China	Chronic hepatitis C virus infection patients	Silymarin/Placebo	2	2,012	600 to 1,260 mg/d	NR	NR	12 to 24 weeks	No	Cochrane	ALT	Yes
(32)	China	Chronic hepatitis B patients	Silymarin and its combination therapy/Placebo	6	603	NR	NR	NR	1 to 3 months	No	Jadad scale	AST, ALT	Yes

T2DM, type 2 diabetes mellitus; NAFLD, non-alcoholic fatty liver disease; RA, rheumatoid arthritis; NASH, non-alcoholic steatohepatitis; NR, not reported.

### 3.3 Quality assessment

The results of the overall methodological quality evaluated using the AMSTAR-2 checklist are presented in Supplementary Table 2. The analysis revealed that only two studies were of moderate quality, while 12 studies were of low quality, and the remaining 26 were of critically low quality. Methodological quality limitations included item two (57.5% of studies failed to register protocols before conducting the review), item three (95% of studies failed to explain the selection of the study designs in the review), item seven (77.5% of studies did not provide a list of excluded studies and justify the exclusions), and item 10 (95% of studies did not report the source of funding for the individual studies) (Supplementary Table 2).

### 3.4 Effect of natural products and dietary interventions on liver enzymes

#### 3.4.1 Natural herbs

The effects of eight natural herbs, including cinnamon, grape, ginseng, nigella sativa, green coffee beans, garlic, green tea, artichoke, saffron, and sumac, as dietary supplements, were analyzed in the included meta-analysis. Compared with placebo, cinnamon supplementation had no significant effect on serum ALT, AST, and ALP levels (51). However, subgroup analyses found that doses of <1,500 mg/day or duration longer than 12 weeks, as well as T2DM, and elderly patients, demonstrated significantly reduced ALT levels (51). The beneficial effects of cinnamon consumption on serum AST levels have been observed in patients with T2DM and a follow-up period of more than 12 weeks (51). Compared with placebo, grape products failed to reduce the serum concentrations of ALT and AST levels (58). However, the subgroup analysis indicated that grape products effectively reduced ALT and AST levels when consumed for ≥12 weeks (58). There was consistent evidence showing that ginseng failed to reduce ALT, AST, GGT, or ALP concentrations compared to placebo, while no significant changes in the overall effect of ginseng supplementation were observed during subgroup analysis (40). Similarly, green coffee bean supplementation failed to affect serum ALT, AST, and ALP levels compared to placebo in adults (45). Compared to placebo, saffron supplementation did not appear to improve AST, ALT, or ALP levels in adult (50).

In non-alcoholic fatty liver disease (NAFLD) patients, nigella sativa significantly reduced AST and ALT levels compared to placebo (54) and was also found to reduce ALP levels while not affecting AST and ALT in adults (44). Similarly, the garlic group depicted a reduction in ALT and AST levels in NAFLD patients compared to the placebo group (66). However, compared to placebo, it significantly reduced AST levels but did no significant affect ALT levels in adults (42). Compared to placebo, the overall effect of green tea supplementation on the ALT, AST, and ALP was non-significant (41), while subgroup analysis demonstrated that it reduced the AST and ALT levels in NAFLD patients, but a slightly significant increase in liver enzymes was observed in healthy subjects (41). Compared to controls, artichokes significantly reduced AST and ALT levels in obese liver patients, but did not affect ALP levels (61). In addition, sumac consumption also had non-significant effects on AST and ALT levels in patients with metabolic syndrome and related disorders (47).

#### 3.4.2 Phytonutrients

The efficacy of phytonutrients, including curcumin, berberine, anthocyanin, silymarin, conjugated linoleic acid, and resveratrol, in modulating liver function was assessed. The results demonstrated that curcumin supplementation reduced serum ALT and AST levels in NAFLD patients compared to placebo (64). Subgroup analysis revealed that only curcumin doses less than 500 mg/day reduced serum liver enzymes (64). Similarly, berberine was found to effectively reduce serum AST and ALT levels in NAFLD patients compared to controls (35), but no such effect was observed when berberine was compared to placebo (39). Betaine does not affect liver enzymes, including ALT, AST, and GGT levels (57). Similar results were observed with anthocyanin supplementation, which did not indicate a significant effect on ALT and AST levels compared to placebo (69), which was found to be not associated with dose and treatment duration (69).

Silymarin has been revealed to have hepatoprotective effects on anti-tubercular drug-induced liver enzymes compared to placebo, which could reduce serum ALT, AST, and ALP levels and significantly improve liver function (38). In patients with hepatitis B, it was found to reduce serum AST and ALT levels as effectively as the hepatoprotective drugs, which were significantly lower in the silymarin combined with hepatoprotective drug group than in the hepatoprotective drug group (32). However, oral silymarin on ALT was not different from placebo in patients suffering from chronic hepatitis C (33). The treatment with silymarin was more efficacious than placebo therapy in decreasing ALT and AST levels in patients with NAFLD (49).

Conjugated linoleic acid (CLA) supplementation failed to alter ALT and AST levels compared to placebo (59). However, the effect of CLA on ALT and AST in the unhealthy group was significant in subgroup analysis (59). Resveratrol supplementation did not significantly affect liver biomarkers, including ALT, ALP, AST, and GGT levels, in adult participants (71). However, the subgroup analysis suggested that ALT and GGT levels were significantly lower in patients with liver disease following resveratrol supplementation (71). And high-dose resveratrol supplementation (>1,000 mg/day) increased ALP and ALT levels in the elderly (≥60 years) (71). In addition, compared to placebo, resveratrol intake did not significantly change ALT, AST, GGT, or ALP levels in NAFLD patients (52).

#### 3.4.3 Micronutrients

The effects of five micronutrients–tocotrienol, vitamin E, vitamin D, chromium, and l-carnitine–were analyzed in this review. The results demonstrated that tocotrienol intake was not associated with serum ALT and AST levels compared to placebo (62), whereas reduced ALT and AST levels were observed with vitamin E compared to placebo (55). Similarly, vitamin D supplementation decreased ALT levels, but no significant changes in AST, ALP, and GGT levels in NAFLD patients were observed, compared to placebo (53). Chromium did not affect AST and ALT levels in T2DM patients compared to placebo (46), while l-carnitine significantly reduced serum ALT, AST, and GGT levels compared to placebo or no treatment (43). Subgroup analysis revealed that l-carnitine supplementation had beneficial effects in lowering enzymes when administered at higher doses (≥2,000 mg/day), longer treatment duration (>12 weeks), and in patients with liver disease (43). In patients with chronic liver disease, l-carnitine significantly reduced ALT and AST levels compared with the control group (65).

#### 3.4.4 Dietary patterns

The effects of six dietary patterns, including high-protein, low-carbohydrate, Mediterranean, calorie-restricted, and soy diets, and nighttime eating habits, were examined in the included meta-analysis. The high protein diet group revealed a significant reduction in AST levels in adults, while ALT levels remained independent (68). Similarly, the low-carbohydrate diet demonstrated non-significant differences in AST and ALT levels in NAFLD patients compared to the low-fat diet (36). In contrast, the Mediterranean diet significantly reduced AST and GGT levels but did not substantially affect ALT levels in adults (67). In NAFLD patients, the Mediterranean and calorie-restricted diet interventions significantly reduced post-intervention ALT levels, and the Mediterranean component intervention had no significant effect (60). In contrast, the Mediterranean, Mediterranean component, and calorie-restricted diets did not impact AST levels (60). Compared to NAFLD controls, the soy diet had no significant effect on AST and ALT levels (56). It was observed that evening snacks could reduce serum AST and ALT levels in patients with cirrhosis (37).

#### 3.4.5 Other interventions

The effects of propolis, polyunsaturated fatty acids, nuts, and non-sweet nutrients on liver enzymes have also been reported. One study showed that propolis intake significantly reduced ALT and AST levels compared to placebo (48). Another study observed that almond intake did not significantly alter ALT, AST and GGT levels, but subgroup analysis indicated significantly reduced ALT levels in unhealthy subjects (63). Compared to placebo or no treatment, polyunsaturated fatty acid supplementation favored NAFLD treatment and GGT levels but failed to reduce ALT and AST levels (34). Similarly, non-nutritional sweetener consumption was reported to non-significantly alter the AST, GGT, or ALT levels compared to placebo (70). However, according to the subgroup analysis, >24 weeks of stevioside intervention significantly reduced ALT levels (70). Moreover, non-nutritional sweeteners have been found to significantly reduce AST levels in T2DM patients (70).

### 3.5 Safety

Six meta-analyses reported on adverse events (32, 33, 38, 54, 64, 68). Gastrointestinal symptoms, including nausea/vomiting, abdominal distension/pain, and anorexia, were the most common. Rash/exanthema, headaches, musculoskeletal pain have been observed in other studies.

### 3.6 Evidence map

**FIGURE 2:**
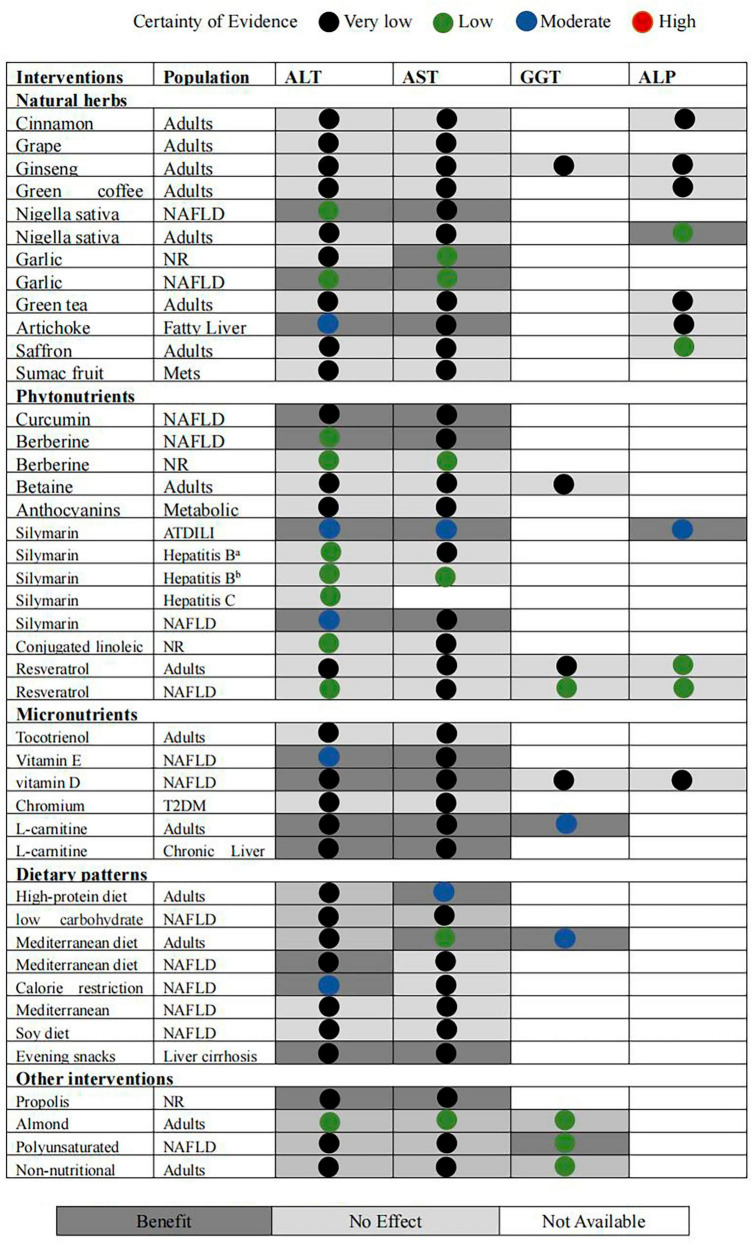
Evidence map of availability and appraisal of certainty of the evidence. *^a^*Silymarin VS protection liver drugs; *^b^*silymarin plus protection liver drugs/protection liver drugs.

The intervention results are summarized in the evidence map displayed in Figure 2. The results demonstrated some evidence regarding nigella sativa, garlic, artichoke, curcumin, silymarin, vitamin E, vitamin D, L-carnitine, and high-protein, Mediterranean, and calorie restriction diets, and evening snacks, propolis, and polyunsaturated fatty acids, which may reduce certain liver enzymes. However, the map also showed that nutritional supplements and dietary patterns did not significantly affect liver enzymes, that the quality of evidence for most interventions ranged from very low to low, and that none of them were supported by high-quality evidence. The evidence quality was generally poor due to the risk of bias, heterogeneity, and imprecision (Supplementary Table 3).

## 4 Discussion

This review presents 40 nutritional supplements and dietary interventions evaluating data from meta-analyses of RCTs, where some evidence was observed stating nigella sativa, garlic, artichoke, curcumin, silymarin, or dietary interventions such as vitamin E, vitamin D, L-carnitine, propolis, polyunsaturated fatty acid, and high-protein, Mediterranean, and calorie restriction diets, and evening snacks may reduce liver enzymes. And it had a good safety profile.

The liver is the largest digestive organ in the body, plays an indispensable role in various physiological mechanisms, and is susceptible to damage by multiple factors, such as metabolites, circulating substances, toxins, and microorganisms (72, 73). Oxidative stress systemic and hepatic inflammation are the main pathological factors associated with developing and progressing liver disorders (51). Several potential mechanisms may elucidate the protective effects on the liver offered by natural products, owing to their antioxidant and anti-inflammatory properties, and, therefore, may benefit liver disorders. Meanwhile, natural products positively affect insulin resistance, decrease fat accumulation in hepatocytes and reduce liver enzymes, especially in patients with T2DM and NAFLD. For example, nigella sativa has high anti-oxidant activity and has been revealed to regenerate pancreatic cells, maintain their integrity and increase insulin secretion (54). Nigella sativa could also reduce inflammatory mediators by increasing the production of prodigiosin E-2 and reducing hepatocyte apoptosis (54). Similarly, S-allyl cysteine, an active ingredient in garlic, exerts potent anti-inflammatory effects by upregulating the peroxisome proliferator-activated receptor, which blocks the proliferation of inflammatory factors, thereby mediating liver enzymes (74). Curcumin has been revealed to inhibit oxidative stress-related inflammation via PI3K/AKT and NF-κB related signaling pathways to alleviate liver injury (75), whereas berberine has also been reported to inhibit oxidative stress and hepatic inflammation, preventing NASH progression (76). Silymarin can decrease metabolic stress in liver cells and inhibit inflammation by reducing the infiltration and activation of macrophages and neutrophils to prevent NASH progression (32). These regulatory mechanisms prevent liver damage, stabilize cell membranes, and reduce cell membrane permeability. The phenolic structure of silymarin allows it to form stable compounds with reactive oxygen species (ROS), which forms the basis of its hepatoprotective and antioxidant properties (77).

The fat-soluble vitamins, including vitamins E and D, are stored and metabolized in the liver. Vitamin E has been clinically proven to improve NAFLD pathology due to its antioxidant effect by reducing steatosis and liver oxidative stress, inflammation, and fibrosis (78). Similarly, vitamin D receptors (VDR) are abundantly expressed in hepatocytes and have been reported to have an exert-inflammatory therapeutic effects on the liver (79). Vitamin D has been found to improve NAFLD and metabolic abnormalities by activating the hepatic VDR, leading to their interaction with HNF4α (80). Our findings reinforced the possible benefits of utilizing vitamins E and D in preventing and treating NAFLD in clinical settings. Moreover, it is well known that disruption of β-oxidation is a significant factor in NAFLD pathogenesis, resulting in fatty acid accumulation within hepatocytes, thereby promoting disease progression. It is possible that the L-carnitine in the transfer of long-chain fatty acids within mitochondria for β-oxidation could potentially contribute to the reduction of ALT and AST levels, particularly in individuals with liver disorders (43).

The development of NAFLD is closely related to diet and lifestyle. It is well supported that different dietary interventions may influence NAFLD pathogenesis and related diseases (81). Many studies have recommended healthy diets and regular physical activities as the primary treatment for NAFLD. Mediterranean diet, as the optimal dietary pattern, has been demonstrated to exert a positive therapeutic effect on liver injury. Our investigation also confirmed a significant reduction in the AST and GGT levels. The Mediterranean diet mainly comprises plant-based foods and fish, with reduced meat and dairy consumption; therefore, it is characterized by a high antioxidant and fiber content, a balanced lipid composition, and a low monosaccharide content in its nutritional composition (82), which is envisaged to reduce hepatic steatosis and metabolic dysfunction in NAFLD patients (83). Other dietary patterns, such as a high-protein diet, can also affect liver enzyme homeostasis. A high-protein diet is associated with satiety, appetite control, and LDL-C and triglyceride levels, suggesting that the diet plays a role in reversing obesity and other chronic diseases. Consequently, the reduction in AST levels we observed in our study may be due to reduced accumulation of triglycerides or alleviated hepatocyte inflammation (84). In addition, our study demonstrated that cirrhotic patients consuming late-night snacks before sleep, providing adequate protein and calories, can reduce protein energy expenditure during early morning hunger (85). The findings of our review are consistent with the evidence that protein intake affects liver function. We also observed that propolis has hepatoprotective effects and reduces AST and ALT levels, mainly mediated by one of its active ingredients, caffeic acid phenethyl ester (86). Similarly, PUFAs have been proven to have a prospective function in NAFLD, which activates the peroxisome proliferator-activated receptor (PPAR), stimulating fatty acid oxidation, and reducing hepatic ROS (87).

There was still considerable room for improvement in the overall methodological quality of the included meta-analyses. First, it has been shown that the availability of registration before a systematic review and meta-analysis affects methodological quality. The systematic review and meta-analysis protocol should be designed and made public before conducting the review to facilitate researchers to reduce implementation and reporting bias. The protocols can be registered on open platforms such as PROSPERO^2^ and OSF REGISTRIES^3^ or made openly available. Second, the included literature is the cornerstone of systematic reviews and meta-analyses, and a comprehensive search strategy guarantees reliability. To improve the accuracy of systematic review and meta-analysis findings, it is recommended that reviewers employ multiple search methods and databases, trace references, search registration platforms, consult relevant field experts, and search gray literature to prevent missing relevant literature. Third, the rationale and reasons for including a particular study type or studies in systematic reviews and meta-analyses should be explained so that reviewers and readers can understand whether the relevant process makes sense. The authors should list potentially excluded literature and mark reasonable reasons to avoid bias in the outcomes. In addition, commercially funded studies are more likely to reach conclusions favoring the sponsor’s product than independently funded studies (88, 89). Therefore, researchers must document the funding source for each study to facilitate judgment of whether financial support gives rise to conflicts of interest that affect the conclusion.

This review has several strengths and limitations. The strengths include meta-analyses based on RCTs while considering dietary interventions and natural products. Because evidence maps were generated from RCTs, this report helps to cover the ‘no evidence zone’ in this domain. This is the first study to comprehensively review the evidence for natural products, nutrients, dietary supplements, and dietary patterns on liver enzymes, accompanied by a wide-ranging and rigorous study design, including a comprehensive quality assessment of the included literature using the latest version of the AMSTAR-2 checklist. However, it must be acknowledged that the AMSTAR-2 checklist and GRADE system are subjective measures that fail to accurately identify the specific methodological and analytical limitations of the literature. Another limitation of this review was that RCTs without conducted meta-analysis were excluded; therefore, the treatment and safety information may not represent the complete results. The quality of systematic review reports needs to be improved to make more confident recommendations, and the methodological rigor of systematic reviews in nutrition interventions is an important field for future research. Future reviews should be rigorously reported by PRISMA guidelines and incorporate established practice methods such as Cochrane guideline.

## 5 Conclusion

This umbrella review of the efficacy of nutritional supplements and dietary interventions on liver enzymes revealed that nigella sativa, garlic, artichoke, curcumin, silymarin, vitamin E, vitamin D, l-carnitine, propolis, PUFAs, high-protein, Mediterranean, calorie restriction diets, and evening snacks reduced liver enzymes levels. The results could help professionals make and modify their recommendations, provide evidence for clinicians, and guide new research to fill evidence gaps.

## Data availability statement

The original contributions presented in this study are included in this article/Supplementary material, further inquiries can be directed to the corresponding author.

## Author contributions

YW: Funding acquisition, Supervision, Writing—review and editing. ZL: Conceptualization, Methodology, Writing—original draft. JW: Formal analysis, Investigation, Methodology, Writing—original draft. YZ: Data curation, Formal analysis, Methodology, Writing—review and editing. JS: Formal analysis, Investigation, Methodology, Writing—original draft.

## Footnotes

1
https://gdt.gradepro.org/app/

2
https://www.crd.york.ac.uk/prospero/

3
https://osf.io/registries?viewonly=
