# Supplementary material for: Natural products and dietary interventions on liver enzymes: an umbrella review and evidence map
Source: Front Nutr. 2024 Feb 2;11:1300860. doi: 10.3389/fnut.2024.1300860 (PMC10869519; doi:10.3389/fnut.2024.1300860)
Supplement: Supplementary file 1 [file Table_1.DOCX]

Supplementary Material

### **Supplementary Table 1. Electronic Database Search Strategies**

**Searches executed 31 March 2023; total number records retrieved: 8043**

| **Pubmed:**  **1974 results：**  (("Alanine Transaminase"[Mesh]) OR (((((Alanine Transaminase[Title/Abstract]) OR (SGPT[Title/Abstract])) OR (ALT[Title/Abstract])) OR (Glutamic Alanine Transaminase[Title/Abstract])) OR (Glutamic Pyruvic Transaminase[Title/Abstract])) OR (Alanine Aminotransferase[Title/Abstract]) OR ("Aspartate Aminotransferases"[Mesh]) OR (((((Aspartate Aminotransferases[Title/Abstract]) OR (SGOT[Title/Abstract])) OR (Glutamate-Aspartate Transaminase[Title/Abstract])) OR (AST[Title/Abstract])) OR (Aspartate Transaminase[Title/Abstract])) OR (Glutamic-Oxaloacetic Transaminase[Title/Abstract]) OR ("Alkaline Phosphatase"[Mesh]) OR (Alkaline Phosphatase[Title/Abstract]) OR (ALP[Title/Abstract]) OR ("gamma-Glutamyltransferase"[Mesh]) OR ((gamma-Glutamyltransferase[Title/Abstract]) OR (GGTP[Title/Abstract])) OR (GGT[Title/Abstract]) OR ((liver function[Title/Abstract]) OR (liver enzymes[Title/Abstract])) OR (liver enzyme[Title/Abstract]) ) AND ((((((Systematic review[Title/Abstract])) OR (Meta analyses[Title/Abstract])) OR (meta analysis[Title/Abstract])) OR (Systematic reviews[Title/Abstract])) OR (("Meta-Analysis" [Publication Type] OR "Meta-Analysis as Topic"[Mesh]) OR ("Systematic Review" [Publication Type] OR "Systematic Reviews as Topic"[Mesh]))) AND("0001/01/01"[PDAT]:"2023/03/31"[PDAT])AND ("Humans"[Mesh]) |
| --- |
| **Embase**  **4509 results：**  ('alanine aminotransferase blood level'/exp OR 'alanine aminotransferase':ti,ab,kw OR sgpt:ti,ab,kw OR alt:ti,ab,kw OR 'glutamic alanine transaminase':ti,ab,kw OR 'glutamic pyruvic transaminase':ti,ab,kw OR 'alanine transaminase':ti,ab,kw OR 'alanine aminotransferase blood level':ti,ab,kw OR 'aspartate aminotransferase'/exp OR 'aspartate aminotransferases':ti,ab,kw OR 'sgot':ti,ab,kw OR 'glutamate-aspartate transaminase':ti,ab,kw OR ast:ti,ab,kw OR 'aspartate transaminase':ti,ab,kw OR 'glutamic-oxaloacetic transaminase':ti,ab,kw OR 'alkaline phosphatase'/exp OR 'alkaline phosphatase':ti,ab,kw OR 'alp':ti,ab,kw OR 'gamma glutamyltransferase'/exp OR 'gamma glutamyltransferase':ti,ab,kw OR ggtp:ti,ab,kw OR ggt:ti,ab,kw OR 'liver function':ti,ab,kw OR 'liver enzymes':ti,ab,kw OR 'liver enzyme':ti,ab,kw) AND ('meta analysis'/exp OR 'systematic review'/exp OR 'systematic review':ti,ab,kw OR 'meta analyses':ti,ab,kw OR 'meta analysis':ti,ab,kw OR 'systematic reviews':ti,ab,kw) AND [humans]/lim NOT ([conference abstract]/lim OR [conference review]/lim OR [editorial]/lim OR [letter]/lim OR [note]/lim OR [short survey]/lim) AND [01-01-0001]/sd NOT [01-04-2023]/sd |
| **Cochrane library**  **1560 results：**  #1 MeSH descriptor: [Alanine Transaminase] explode all trees  #2 (“Alanine Transaminase”):ti,ab,kw OR (SGPT):ti,ab,kw OR (ALT):ti,ab,kw OR (“Glutamic Alanine Transaminase”):ti,ab,kw OR (“Glutamic Pyruvic Transaminase”):ti,ab,kw OR (“Alanine Aminotransferase”):ti,ab,kw (Word variations have been searched) in Cochrane Reviews  #3 #1 OR #2  #4 MeSH descriptor: [Aspartate Aminotransferases] explode all trees  #5 (“Aspartate Aminotransferases”):ti,ab,kw OR (SGOT):ti,ab,kw OR (“Glutamate-Aspartate Transaminase”):ti,ab,kw OR (AST):ti,ab,kw OR (“Aspartate Transaminase”):ti,ab,kw OR (“Glutamic-Oxaloacetic Transaminase”):ti,ab,kw (Word variations have been searched) in Cochrane Reviews  #6 #4 OR #5  #7 MeSH descriptor: [Alkaline Phosphatase] explode all trees  #8 (“Alkaline Phosphatase”) :ti,ab,kw OR (ALP) :ti,ab,kw  #9 #7 OR #8  #10 MeSH descriptor: [gamma-Glutamyltransferase] explode all trees  #11 (“gamma-Glutamyltransferase”) :ti,ab,kw OR (GGTP) :ti,ab,kw OR (GGT) :ti,ab,kw  #12 #10 OR #11  #13 (“liver function”) :ti,ab,kw OR (“liver enzymes”) :ti,ab,kw OR (“liver enzyme”) :ti,ab,kw  #14 #3 OR #6 OR #9 OR #12 OR #13 with Cochrane Library publication date Between Jan 0001 and Mar 2023, in Cochrane Reviews |
|  |
|  |
|  |
|  |
|  |
|  |
|  |
|  |
|  |
|  |
|  |
|  |
|  |
