# Supplementary material for: Natural products and dietary interventions on liver enzymes: an umbrella review and evidence map
Source: Front Nutr. 2024 Feb 2;11:1300860. doi: 10.3389/fnut.2024.1300860 (PMC10869519; doi:10.3389/fnut.2024.1300860)
Supplement: Supplementary file 2 [file Table_2.DOCX]

Supplementary Material

**Supplementary Table S2 Results of methodological quality**

| **First author, year** | **Item1** | **Item2** | **Item3** | **Item4** | **Item5** | **Item6** | **Item7** | **Item8** | **Item9** | **Item10** | **Item11** | **Item12** | **Item13** | **Item14** | **Item15** | **Item16** | **Overall quality** |
| --- | --- | --- | --- | --- | --- | --- | --- | --- | --- | --- | --- | --- | --- | --- | --- | --- | --- |
| Mousavi, 2021 | Y | Y | Y | P | Y | Y | N | Y | Y | N | Y | Y | Y | Y | Y | Y | Low |
| Ghaffar, 2022 | Y | P | N | P | Y | Y | N | Y | Y | N | Y | Y | Y | Y | Y | Y | Low |
| Ghavami, 2020 | Y | N | N | P | Y | Y | N | Y | Y | N | Y | Y | Y | Y | Y | Y | Critically low |
| Asbaghi, 2021 | N | N | N | P | Y | Y | N | N | Y | N | N | Y | N | Y | Y | Y | Critically low |
| Tang, 2021 | Y | N | N | P | Y | Y | N | Y | Y | N | Y | Y | Y | Y | Y | Y | Critically low |
| Razmpoosh, 2020 | Y | Y | N | P | Y | Y | Y | Y | Y | N | Y | Y | Y | Y | Y | Y | Low |
| Panjeshahin, 2020 | N | N | N | P | N | Y | N | Y | Y | N | Y | Y | Y | Y | Y | Y | Critically low |
| Rastkar, 2022 | Y | Y | N | P | Y | Y | N | Y | Y | N | Y | N | N | N | N | Y | Critically low |
| Mahmoodi, 2020 | Y | N | N | P | Y | Y | Y | Y | Y | N | N | Y | N | Y | Y | Y | Critically low |
| Kamel, 2022 | Y | Y | N | P | Y | Y | N | Y | Y | N | Y | Y | Y | Y | Y | Y | Low |
| Karimi, 2021 | Y | N | N | P | Y | Y | N | Y | Y | N | Y | Y | Y | Y | Y | Y | Critically low |
| Ngu, 2022 | Y | Y | N | P | Y | Y | Y | Y | Y | Y | Y | Y | Y | Y | Y | Y | Moderate |
| Wei, 2016 | Y | N | N | P | Y | Y | N | P | Y | N | Y | Y | Y | Y | Y | Y | Critically low |
| Asbaghi, 2020 | N | N | N | P | N | Y | N | Y | Y | N | Y | N | Y | Y | N | Y | Critically low |
| Ashtary-Larky, 2022 | Y | N | N | P | Y | Y | P | N | Y | N | Y | Y | Y | Y | Y | Y | Low |
| Zhu, 2022 | Y | P | N | P | Y | Y | N | Y | Y | N | Y | Y | Y | Y | Y | Y | Low |
| Tao, 2019 | Y | N | N | P | Y | Y | Y | Y | Y | N | Y | Y | Y | Y | Y | Y | low |
| Wei, 2013 | Y | N | N | P | Y | Y | N | P | P | N | Y | N | N | Y | N | Y | Critically low |
| Yang, 2014 | Y | N | N | P | Y | Y | Y | P | Y | N | Y | N | Y | N | N | Y | Critically low |
| [Kalopitas](https://pubmed.ncbi.nlm.nih.gov/?term=Kalopitas+G&cauthor_id=33418491), 2021 | Y | Y | N | P | Y | Y | N | Y | Y | N | Y | Y | Y | Y | N | Y | Critically low |
| Haghighat, 2022 | Y | Y | N | P | Y | Y | N | Y | Y | N | Y | Y | Y | Y | Y | Y | Critically low |
| Soltani, 2023 | Y | Y | Y | P | Y | Y | N | Y | Y | Y | Y | Y | Y | Y | Y | Y | Low |
| [Rafiee](https://pubmed.ncbi.nlm.nih.gov/?term=Rafiee+S&cauthor_id=33321448), 2021 | Y | N | N | P | Y | Y | N | Y | Y | N | Y | N | N | Y | Y | Y | Critically low |
| Li, 2022 | Y | N | Y | P | N | Y | N | Y | Y | N | Y | N | Y | Y | N | Y | Critically low |
| Vadarlis, 2021 | Y | Y | N | P | Y | Y | N | P | Y | N | N | Y | Y | Y | N | Y | Critically low |
| Rezaei, 2021 | Y | N | N | P | Y | Y | N | P | Y | N | Y | Y | Y | Y | Y | Y | Critically low |
| Asbaghi, 2021 | Y | N | N | P | N | Y | Y | Y | Y | N | Y | Y | N | Y | Y | Y | Critically low |
| Pirmadah, 2020 | Y | Y | N | P | Y | Y | Y | Y | Y | N | Y | Y | Y | Y | Y | Y | Moderate |
| Oh, 2022 | Y | Y | N | P | Y | Y | N | Y | Y | N | Y | Y | Y | Y | Y | Y | Low |
| Zhang, 2022 | Y | N | N | P | Y | Y | N | P | Y | N | Y | Y | Y | Y | Y | Y | Critically low |
| Ahn, 2019 | Y | N | N | P | Y | Y | N | Y | N | N | Y | N | Y | N | N | Y | Critically low |
| Sangouni, 2022 | Y | Y | N | P | Y | Y | N | Y | Y | N | Y | Y | Y | Y | Y | Y | Low |
| Haigh, 2022 | Y | Y | N | P | Y | N | Y | Y | P | N | Y | Y | Y | Y | N | Y | Low |
| Xiong, 2021 | Y | N | N | P | Y | Y | N | Y | P | N | Y | Y | N | Y | N | Y | Critically low |
| Chen, 2019 | Y | N | N | P | N | Y | N | N | Y | N | Y | N | Y | Y | Y | Y | Critically low |
| Hallajzadeh, 2021 | N | N | N | N | N | Y | N | Y | N | N | Y | Y | Y | Y | N | Y | Critically low |
| Mostafa Qorbani, 2022 | Y | N | N | P | Y | Y | N | Y | Y | N | Y | Y | Y | Y | N | Y | Critically low |
| Lu, 2016 | Y | N | N | P | Y | Y | N | N | P | N | Y | N | N | N | Y | Y | Critically low |
| Golzan, 2023 | Y | Y | Y | P | Y | Y | N | Y | Y | N | Y | Y | Y | Y | Y | Y | Low |
| Ghafouri, 2021 | Y | Y | N | P | Y | Y | N | N | Y | N | N | N | N | Y | N | Y | Critically low |
| **Y (%)** | 36(90%) | 15(37.5%) | 2(5%) |  | 34(85%) | 39(97.5%) | 8(20%) | 29(72.5%) | 34(85%) | 2(5%) | 36(90%) | 30(75%) | 31(77.5%) | 36(90%) | 27(67.5%) | 40(100%) |  |
| **PY (%)** |  | 2(5%) |  | 39(97.5%) |  |  | 1(2.5%) | 6(15%) | 3(7.5%) |  |  |  |  |  |  |  |  |
| N (%) | 4(10%) | 23(57.5%) | 38(95%) | 1(2.5%) | 6(15%) | 1(2.5%) | 31(77.5%) | 5(12.5%) | 3(7.5%) | 38(95%) | 4(10%) | 10(25%) | 9(22.5%) | 4(10%) | 13(32.5%) |  |  |

Note Y: yes; N: no; P: partial yes. **Item 1:** Did the research questions and inclusion criteria for the review include the components of PICO?; **Item 2:** Did the report of the review contain an explicit statement that the review methods were established prior to the conduct of the review and did the report justify any significant deviations from the protocol?; **Item 3:** Did the review authors explain their selection of the study designs for inclusion in the review?; **Item 4:** Did the review authors use a comprehensive literature search strategy?; **Item 5:** Did the review authors perform study selection in duplicate?; **Item 6:** Did the review authors perform data extraction in duplicate?; **Item 7:** Did the review authors provide a list of excluded studies and justify the exclusions?; **Item 8:** Did the review authors describe the included studies in adequate detail?; **Item 9:** Did the review authors use a satisfactory technique for assessing the risk of bias (RoB) in individual studies that were included in the review?; **Item 10:** Did the review authors report on the sources of funding for the studies included in the review?; **Item 11:** If meta-analysis was performed did the review authors use appropriate methods for statistical combination of results?; **Item 12:** If meta-analysis was performed, did the review authors assess the potential impact of RoB in individual studies on the results of the meta-analysis or other evidence synthesis?; **Item 13:** Did the review authors account for RoB in individual studies when interpreting/discussing the results of the review?; **Item 14:** Did the review authors provide a satisfactory explanation for, and discussion of, any heterogeneity observed in the results of the review?; **Item 15:** If they performed quantitative synthesis did the review authors carry out an adequate investigation of publication bias (small study bias) and discuss its likely impact on the results of the review?; **Item 16:** Did the review authors report any potential sources of conflict of interest, including any funding they received for conducting the review?.

References:

Ahn J, Jun, DW, Lee, HY, Moon, JH. 2019. Critical appraisal for low-carbohydrate diet in nonalcoholic fatty liver disease: Review and meta-analyses. Clin Nutr. 38: 2023-2030.

Asbaghi O, Ghanbari, N, Shekari, M, Reiner, Ž, Amirani, E, Hallajzadeh, J, et al. 2020. The effect of berberine supplementation on obesity parameters, inflammation and liver function enzymes: A systematic review and meta-analysis of randomized controlled trials. Clin Nutr ESPEN. 38: 43-49.

Asbaghi O, Kashkooli, S, Mardani, M, Rezaei Kelishadi, M, Fry, H, Kazemi, M, et al. 2021. Effect of green coffee bean extract supplementation on liver function and inflammatory biomarkers: A meta-analysis of randomized clinical trials. Complement Ther Clin Pract. 43: 101349.

Asbaghi O, Naeini, F, Ashtary-Larky, D, Kaviani, M, Rezaei Kelishadi, M, Eslampour, E, et al. 2021. Effects of chromium supplementation on blood pressure, body mass index, liver function enzymes and malondialdehyde in patients with type 2 diabetes: A systematic review and dose-response meta-analysis of randomized controlled trials. Complement Ther Med. 60: 102755.

Ashtary-Larky D, Bagheri, R, Ghanavati, M, Asbaghi, O, Tinsley, GM, Mombaini, D, et al. 2022. Effects of betaine supplementation on cardiovascular markers: A systematic review and Meta-analysis. Crit Rev Food Sci Nutr. 62: 6516-6533.

Chen C-J, Wang, L-C, Kuo, H-T, Fang, Y-C, Lee, H-F. 2019. Significant effects of late evening snack on liver functions in patients with liver cirrhosis: A meta-analysis of randomized controlled trials. J Gastroenterol Hepatol. 34: 1143-1152.

Ghaffar S, Naqvi, MA, Fayyaz, A, Abid, MK, Khayitov, KN, Jalil, AT, et al. 2022. What is the influence of grape products on liver enzymes? A systematic review and meta-analysis of randomized controlled trials. Complement Ther Med. 69: 102845.

Ghafouri A, Estêvão, MD, Alibakhshi, P, Pizarro, AB, Kashani, AF, Persad, E, et al. 2021. Sumac fruit supplementation improve glycemic parameters in patients with metabolic syndrome and related disorders: A systematic review and meta-analysis. Phytomedicine. 90: 153661.

Ghavami A, Ziaei, R, Foshati, S, Hojati Kermani, MA, Zare, M, Amani, R. 2020. Benefits and harms of ginseng supplementation on liver function? A systematic review and meta-analysis. Complement Ther Clin Pract. 39: 101173.

Golzan SA, Movahedian, M, Haghighat, N, Asbaghi, O, Hekmatdoost, A. 2023. Association between non-nutritive sweetener consumption and liver enzyme levels in adults: a systematic review and meta-analysis of randomized clinical trials. Nutr Rev.

Haghighat N, Shimi, G, Shiraseb, F, Karbasi, A, Nadery, M, Ashtary-Larky, D, et al. 2022. The effects of conjugated linoleic acid supplementation on liver function enzymes and malondialdehyde in adults: A GRADE-assessed systematic review and dose-response meta-analysis. Pharmacol Res. 186: 106518.

Haigh L, Kirk, C, El Gendy, K, Gallacher, J, Errington, L, Mathers, JC, et al. 2022. The effectiveness and acceptability of Mediterranean diet and calorie restriction in non-alcoholic fatty liver disease (NAFLD): A systematic review and meta-analysis. Clin Nutr. 41: 1913-1931.

Hallajzadeh J, Milajerdi, A, Amirani, E, Attari, VE, Maghsoudi, H, Mirhashemi, SM. 2021. Effects of propolis supplementation on glycemic status, lipid profiles, inflammation and oxidative stress, liver enzymes, and body weight: a systematic review and meta-analysis of randomized controlled clinical trials. J Diabetes Metab Disord. 20: 831-843.

Kalopitas G, Antza, C, Doundoulakis, I, Siargkas, A, Kouroumalis, E, Germanidis, G, et al. 2021. Impact of Silymarin in individuals with nonalcoholic fatty liver disease: A systematic review and meta-analysis. Nutrition. 83: 111092.

Kamel AM, Farag, MA. 2022. Therapeutic Potential of Artichoke in the Treatment of Fatty Liver: A Systematic Review and Meta-Analysis. J Med Food. 25: 931-942.

Karimi E, Farrokhzad, A, Darand, M, Arab, A. 2021. The Effect of Saffron Consumption on Liver Function: A Systematic Review and Meta-Analysis of Randomized Controlled Clinical Trials. Complement Med Res. 28: 453-462.

Li F, Xu, B, Soltanieh, S, Zanghelini, F, Abu-Zaid, A, Sun, J. 2022. The effects of tocotrienols intake on obesity, blood pressure, inflammation, liver and glucose biomarkers: a meta-analysis of randomized controlled trials. Crit Rev Food Sci Nutr. 62: 7154-7167.

Lu W, Li, S, Li, J, Wang, J, Zhang, R, Zhou, Y, et al. 2016. Effects of Omega-3 Fatty Acid in Nonalcoholic Fatty Liver Disease: A Meta-Analysis. Gastroenterol Res Pract. 2016: 1459790.

Mahmoodi M, Hosseini, R, Kazemi, A, Ofori-Asenso, R, Mazidi, M, Mazloomi, SM. 2020. Effects of green tea or green tea catechin on liver enzymes in healthy individuals and people with nonalcoholic fatty liver disease: A systematic review and meta-analysis of randomized clinical trials. Phytother Res. 34: 1587-1598.

Morvaridzadeh M, Qorbani, M, Shokati Eshkiki, Z, Estêvão, MD, Mohammadi Ganjaroudi, N, Toupchian, O, et al. 2022. The effect of almond intake on cardiometabolic risk factors, inflammatory markers, and liver enzymes: A systematic review and meta-analysis. Phytother Res. 36: 4325-4344.

Mousavi SM, Jayedi, A, Bagheri, A, Zargarzadeh, N, Wong, A, Persad, E, et al. 2021. What is the influence of cinnamon supplementation on liver enzymes? A systematic review and meta-analysis of randomized controlled trials. Phytother Res. 35: 5634-5646.

Ngu MH, Norhayati, MN, Rosnani, Z, Zulkifli, MM. 2022. Curcumin as adjuvant treatment in patients with non-alcoholic fatty liver (NAFLD) disease: A systematic review and meta-analysis. Complement Ther Med. 68: 102843.

Oh H, Park, CH, Jun, DW. 2022. Impact of l-Carnitine Supplementation on Liver Enzyme Normalization in Patients with Chronic Liver Disease: A Meta-Analysis of Randomized Trials. J Pers Med. 12.

Panjeshahin A, Mollahosseini, M, Panbehkar-Jouybari, M, Kaviani, M, Mirzavandi, F, Hosseinzadeh, M. 2020. Effects of garlic supplementation on liver enzymes: A systematic review and meta-analysis of randomized controlled trials. Phytother Res. 34: 1947-1955.

Pirmadah F, Ramezani-Jolfaie, N, Mohammadi, M, Talenezhad, N, Clark, CCT, Salehi-Abargouei, A. 2020. Does L-carnitine supplementation affect serum levels of enzymes mainly produced by liver? A systematic review and meta-analysis of randomized controlled clinical trials. Eur J Nutr. 59: 1767-1783.

Rafiee S, Mohammadi, H, Ghavami, A, Sadeghi, E, Safari, Z, Askari, G. 2021. Efficacy of resveratrol supplementation in patients with nonalcoholic fatty liver disease: A systematic review and meta-analysis of clinical trials. Complement Ther Clin Pract. 42: 101281.

Rastkar M, Nikniaz, L, Abbasalizad Farhangi, M, Nikniaz, Z. 2022. Systematic review and meta-analysis of the effect of garlic in patients with non-alcoholic fatty liver disease. Indian J Gastroenterol. 41: 548-557.

Razmpoosh E, Safi, S, Abdollahi, N, Nadjarzadeh, A, Nazari, M, Fallahzadeh, H, et al. 2020. The effect of Nigella sativa on the measures of liver and kidney parameters: A systematic review and meta-analysis of randomized-controlled trials. Pharmacol Res. 156: 104767.

Rezaei S, Tabrizi, R, Nowrouzi-Sohrabi, P, Jalali, M, Shabani-Borujeni, M, Modaresi, S, et al. 2021. The Effects of Vitamin D Supplementation on Anthropometric and Biochemical Indices in Patients With Non-alcoholic Fatty Liver Disease: A Systematic Review and Meta-analysis. Front Pharmacol. 12: 732496.

Sangouni AA, Hassani Zadeh, S, Mozaffari-Khosravi, H, Hosseinzadeh, M. 2022. Effect of Mediterranean diet on liver enzymes: a systematic review and meta-analysis of randomised controlled trials. Br J Nutr. 128: 1231-1239.

Soltani S, Sharifi-Zahabi, E, Sangsefidi, ZS, Ahmadi Vasmehjani, A, Meshkini, F, Clayton, ZS, et al. 2023. The effect of resveratrol supplementation on biomarkers of liver health: A systematic review and meta-analysis of randomized controlled trials. Phytother Res. 37: 1153-1166.

Tang G, Zhang, L, Tao, J, Wei, Z. 2021. Effect of Nigella sativa in the treatment of nonalcoholic fatty liver disease: A systematic review and meta-analysis of randomized controlled trials. Phytother Res. 35: 4183-4193.

Tao L, Qu, X, Zhang, Y, Song, Y, Zhang, S-X. 2019. Prophylactic Therapy of Silymarin (Milk Thistle) on Antituberculosis Drug-Induced Liver Injury: A Meta-Analysis of Randomized Controlled Trials. Can J Gastroenterol Hepatol. 2019: 3192351.

Vadarlis A, Antza, C, Bakaloudi, DR, Doundoulakis, I, Kalopitas, G, Samara, M, et al. 2021. Systematic review with meta-analysis: The effect of vitamin E supplementation in adult patients with non-alcoholic fatty liver disease. J Gastroenterol Hepatol. 36: 311-319.

Wei F, Liu, SK, Liu, XY, Li, ZJ, Li, B, Zhou, YL, et al. 2013. Meta-analysis: silymarin and its combination therapy for the treatment of chronic hepatitis B. Eur J Clin Microbiol Infect Dis. 32: 657-669.

Wei X, Wang, C, Hao, S, Song, H, Yang, L. 2016. The Therapeutic Effect of Berberine in the Treatment of Nonalcoholic Fatty Liver Disease: A Meta-Analysis. Evid Based Complement Alternat Med. 2016: 3593951.

Xiong P, Zhu, Y-F. 2021. Soy diet for nonalcoholic fatty liver disease: A meta-analysis of randomized controlled trials. Medicine (Baltimore). 100: e25817.

Yang Z, Zhuang, L, Lu, Y, Xu, Q, Chen, X. 2014. Effects and tolerance of silymarin (milk thistle) in chronic hepatitis C virus infection patients: a meta-analysis of randomized controlled trials. Biomed Res Int. 2014: 941085.

Zhang Y, Chen, X, Allison, DB, Xun, P. 2022. Efficacy and safety of a specific commercial high-protein meal-replacement product line in weight management: meta-analysis of randomized controlled trials. Crit Rev Food Sci Nutr. 62: 798-809.

Zhou F, She, W, He, L, Zhu, J, Gu, L. 2022. The effect of anthocyanins supplementation on liver enzymes among patients with metabolic disorders: A systematic review and meta-analysis of randomized clinical trials. Phytother Res. 36: 53-61.
