# Supplementary material for: Natural products and dietary interventions on liver enzymes: an umbrella review and evidence map
Source: Front Nutr. 2024 Feb 2;11:1300860. doi: 10.3389/fnut.2024.1300860 (PMC10869519; doi:10.3389/fnut.2024.1300860)
Supplement: Supplementary file 3 [file Table_3.DOCX]

Supplementary Material

**Supplementary Table S3 GRADE profile of natural products and dietary interventions on liver enzymes**

| **Reference** | **Outcomes** | **Population** | **Interventions/ comparators** | **Number of RCTs** | **Sample size** | **Effect metrics** | **Estimates** | **95%CI** | **I^2^** | **P value** | **Risk of bias** | **Inconsistency** | **Indirection** | **Imprecision** | **publication bias** | **GRADE level** |
| --- | --- | --- | --- | --- | --- | --- | --- | --- | --- | --- | --- | --- | --- | --- | --- | --- |
| Mousavi, 2021 | ALT | Adults | Cinnamon/Placebo | 7 | 256(133/123) | Random/MD | -4.54 | -11.16, 2.08 | 90.1% | < 0.001 | Serious ^a^ | Very serious ^b^ | Not serious | Very serious ^c, d^ | Not serious | **⨁◯◯◯**  **Very low** |
|  | AST | Adults | Cinnamon/Placebo | 6 | 242(126/116) | Random/MD | ­-2.60 | -6.70, 1.50 | 77.3% | < 0.001 | Serious ^a^ | Very serious ^b^ | Not serious | Very serious ^c, d^ | Not serious | **⨁◯◯◯**  **Very low** |
|  | ALP | Adults | Cinnamon/Placebo | 3 | 53(28/25) | Random/MD | 4.22 | -2.84, 11.27 | 0.0% | 0.81 | Serious ^a^ | Not serious | Not serious | Very serious ^c, d^ | Not mention | **⨁◯◯◯**  **Very low** |
| Ghaffar, 2022 | ALT | Adults | Grape products /Placebo | 8 | NR | Random/WMD | -2.04 | -5.50, 1.42 | 72.5 % | P < 0.001 | Serious ^a^ | Serious ^e^ | Not serious | Very serious ^c, d^ | Not serious | **⨁◯◯◯**  **Very low** |
|  | AST | Adults | Grape products /Placebo | 7 | NR | Random/WMD | -1.40 | -3.80, 0.99 | 76.0 % | P˂0.001 | Serious ^a^ | Very serious ^b^ | Not serious | Very serious ^c, d^ | Not serious | **⨁◯◯◯**  **Very low** |
| Ghavami,2020 | ALT | Adults | Ginseng/Placebo | 14 | 992(494 /498) | Random/WMD | 0.04 | -1.80, 1.89 | 49.3% | 0.007 | Serious ^a^ | Not serious | Not serious | Serious ^d^ | Not serious | **⨁◯◯◯**  **Very low** |
|  | AST | Adults | Ginseng/Placebo | 12 | 876 (436 /440) | Random/WMD | 0.16 | -1.19, 1.52 | 53.6% | 0.004 | Serious ^a^ | Serious ^e^ | Not serious | Serious ^d^ | Not serious | **⨁◯◯◯**  **Very low** |
|  | ALP | Adults | Ginseng/Placebo | 4 | 382 (192 /190) | Random/WMD | -0.03 | -0.23, 0.17 | 0.0% | 0.998 | Serious ^a^ | Not serious | Not serious | Serious ^d^ | Not serious | **⨁◯◯◯**  **Very low** |
|  | GGT | Adults | Ginseng/Placebo | 6 | 609 (305 /304) | Random/WMD | 1.04 | -0.69, 2.7 | 0.0% | 0.902 | Serious ^a^ | Not serious | Not serious | Serious ^d^ | Not serious | **⨁◯◯◯**  **Very low** |
| Asbaghi, 2021 | AST | Adults | Green coffee bean extract/Placebo | 7 | NR | Random/WMD | 0.03 | -0.85, 0.80 | 0% | 0.627 | Serious ^a^ | Not serious | Not serious | Serious ^d^ | Not serious | **⨁◯◯◯**  **Very low** |
|  | ALT | Adults | Green coffee bean extract/Placebo | 7 | NR | Random/WMD | 0.03 | -3.48, 3.41 | 92.9% | 0.000 | Serious ^a^ | Very serious ^b^ | Not serious | Serious ^d^ | Not serious | **⨁◯◯◯**  **Very low** |
|  | ALP | Adults | Green coffee bean extract/Placebo | 4 | NR | Random/WMD | 12.22 | -7.82, 32.26 | 84.0% | 0.000 | Serious ^a^ | Very serious ^b^ | Not serious | Serious ^d^ | Not serious | **⨁◯◯◯**  **Very low** |
| Tang, 2021 | AST | NAFLD | Nigella sativa/Placebo | 5 | 358(179/179) | Random/MD | -12.00 | −19.38, −4.62 | 90% | < 0.00001 | Serious ^a^ | Very serious ^b^ | Not serious | Not serious | Not serious | **⨁◯◯◯**  **Very low** |
|  | ALT | NAFLD | Nigella sativa/Placebo | 5 | 358(179/179) | Random/MD | -11.23 | −21.41, −1.05 | 96% | < 0.00001 | Serious ^a^ | Very serious ^b^ | Not serious | Not serious | Not serious | **⨁⨁◯◯**  **Low** |
| Razmpoosh, 2020 | ALT | Adults | Nigella sativa/Placebo | 15 | 1119(563/556) | Random/WMD | -0.074 | −0.682, 0.534 | 38.4 % | 0.065 | Serious ^a^ | Not serious | Not serious | Serious ^d^ | Not serious | **⨁◯◯◯**  **Very low** |
|  | AST | Adults | Nigella sativa/Placebo | 13 | 846(424/422) | Random/WMD | −0.539 | −1.715, 0.636 | 86.3% | < 0.000 | Serious ^a^ | Very serious ^b^ | Not serious | Serious ^d^ | Not serious | **⨁◯◯◯**  **Very low** |
|  | ALP | Adults | Nigella sativa/Placebo | 9 | 710(352/358) | Random/WMD | -10.825 | −19.658, −1.992 | 75.7 % | < 0.000 | Serious ^a^ | Very serious ^b^ | Not serious | Not serious | Not serious | **⨁⨁◯◯**  **Low** |
| Panjeshahin,2020 | AST | NR | Garlic/Placebo | 6 | 301(NR) | Random/ Hedges' g | −0.36 | −0.72, −0.004 | 56.4% | 0.043 | Serious ^a^ | Serious ^e^ | Not serious | Not serious | Not serious | **⨁⨁◯◯**  **Low** |
|  | ALT | NR | Garlic/Placebo | 6 | 301(NR) | Random/ Hedges' g | −0.22 | −0.64, 0.20 | 69.1% | 0.006 | Serious ^a^ | Serious ^e^ | Not serious | Serious ^d^ | Not serious | **⨁◯◯◯**  **Very low** |
| Rastkar, 2022 | AST | NAFLD | Garlic/Placebo | 2 | 186 | Random/SMD | 0.59 | 0.27, 0.85 | 0.000 | 0.70 | Serious ^a^ | Not serious | Not serious | Serious ^c^ | Not mention | **⨁⨁◯◯**  **Low** |
|  | ALT | NAFLD | Garlic/Placebo | 2 | 186 | Random/SMD | 0.60 | 0.31, 0.90 | 0.000 | 0.96 | Serious ^a^ | Not serious | Not serious | Serious ^c^ | Not mention | **⨁⨁◯◯**  **Low** |
| Mahmoodi, 2020 | ALT | Adults | Green tea/Placebo | 15 | NR | NR/SMD | −0.17 | −0.42, 0.08 | 78.5% | <0.001 | Serious ^a^ | Very serious ^b^ | Not serious | Serious ^d^ | Serious ^f^ | **⨁◯◯◯**  **Very low** |
|  | AST | Adults | Green tea/Placebo | 13 | NR | NR/SMD | −0.07 | −0.43, 0.29 | 88.3% | <0.001 | Serious ^a^ | Very serious ^b^ | Not serious | Serious ^d^ | Not serious | **⨁◯◯◯**  **Very low** |
|  | ALP | Adults | Green tea/Placebo | 6 | NR | NR/SMD | −0.17 | −0.45, 0.1 | 68.0% | 0.008 | Serious ^a^ | Serious ^e^ | Not serious | Serious ^d^ | Not serious | **⨁◯◯◯**  **Very low** |
| Kamel,2022 | ALT | Fatty Liver | Artichoke/Control | 5 | 333(172/161) | Random/SMD | 1.11 | 0.79,1.43 | 44% | 0.13 | Serious ^a^ | Not serious | Not serious | Not serious | Not serious | **⨁⨁⨁◯**  **Moderate** |
|  | AST | Fatty Liver | Artichoke/Control | 5 | 333(172/161) | Random/SMD | 1.01 | 0.52,1.51 | 77% | <0.01 | Serious ^a^ | Very serious ^b^ | Not serious | Not serious | Not serious | **⨁◯◯◯**  **Very low** |
|  | ALP | Fatty Liver | Artichoke/Control | 3 | 173(92/81) | Random/SMD | 1.18 | -0.19,2.55 | 93% | <0.01 | Serious ^a^ | Very serious ^b^ | Not serious | Serious ^d^ | Not serious | **⨁◯◯◯**  **Very low** |
| Karimi,2021 | AST | Adults | Saffron/Placebo | 11 | NR | Random/WMD | 0.23 | –2.22, 2.69 | 74.0% | <0.001 | Serious ^a^ | Serious ^e^ | Not serious | Serious ^d^ | Not serious | **⨁◯◯◯**  **Very low** |
|  | ALT | Adults | Saffron/Placebo | 7 | NR | Random/WMD | -1.49 | –3.84, 0.86 | 60.2% | 0.005 | Serious ^a^ | Serious ^e^ | Not serious | Serious ^d^ | Not serious | **⨁◯◯◯**  **Very low** |
|  | ALP | Adults | Saffron/Placebo | 6 | 323(NR) | Random/WMD | -0.70 | –11.35, 9.95 | 40.8% | 0.119 | Serious ^a^ | Not serious | Not serious | Serious ^d^ | Not serious | **⨁⨁◯◯**  **Low** |
| Ghafouri,2021 | AST | Mets and related disorders | Sumac fruit/Placebo | 2 | 150(NR) | Random/SMD | -0.41 | -1.54, 0.72 | 91.4% | 0.001 | Serious ^a^ | Very serious ^b^ | Not serious | Very serious ^c, d^ | Not mention | **⨁◯◯◯**  **Very low** |
|  | ALT | Mets and related disorders | Sumac fruit/Placebo | 2 | 150(NR) | Random/SMD | -0.66 | -2.11, 0.79 | 94.5% | <0.000 | Serious ^a^ | Very serious ^b^ | Not serious | Very serious ^c, d^ | Not mention | **⨁◯◯◯**  **Very low** |
| Ngu,2022 | AST | NAFLD | Curcumin/Placebo | 14 | 792(401/391) | Random/MD | -4.00 | -5.72, -2.28 | 82% | <0.001 | Serious ^a^ | Very serious ^b^ | Not serious | Not serious | Not serious | **⨁◯◯◯**  **Very low** |
|  | ALT | NAFLD | Curcumin/Placebo | 13 | 750(380/370) | Random/MD | -7.02 | -9.83, -4.20 | 83% | < 0.001 | Serious ^a^ | Very serious ^b^ | Not serious | Not serious | Not serious | **⨁◯◯◯**  **Very low** |
| Wei,2016 | ALT | NAFLD | Berberine/ lifestyle intervention, other medicines | 5 | 457(209/248) | Random/MD | -7.37 | -12.31, -2.42 | 73% | 0.003 | Serious ^a^ | Serious ^e^ | Not serious | Not serious | Not mention | **⨁⨁◯◯**  **Low** |
|  | AST | NAFLD | Berberine/ lifestyle intervention, other medicines | 6 | 457(209/248) | Random/MD | -4.99 | -8.60, -1.38 | 76% | 0.007 | Serious ^a^ | Very serious ^b^ | Not serious | Not serious | Not mention | **⨁◯◯◯**  **Very low** |
| Asbaghi,2020 | AST | NR | Berberine/Placebo | 3 | NR | Fixed/WMD | -0.87 | -2.56,0.82 | 0% | 0.476 | Serious ^a^ | Not serious | Not serious | Serious ^d^ | Not mention | **⨁⨁◯◯**  **Low** |
|  | ALT | NR | Berberine/Placebo | 4 | NR | Fixed/WMD | -1.66 | -3.98,0.65 | 30.5% | 0.22 | Serious ^a^ | Not serious | Not serious | Serious ^d^ | Not mention | **⨁⨁◯◯**  **Low** |
| Ashtary-Larky, 2022 | ALT | Adults | Betaine/ Control | 4 | 152(78/74) | Random/MD | 0.45 | -2.98, 3.88 | 0.0% | 0.415 | Serious ^a^ | Not serious | Not serious | Very serious ^c, d^ | Not serious | **⨁◯◯◯**  **Very low** |
|  | AST | Adults | Betaine/ Control | 3 | 104(53/51) | Random/MD | -0.52 | -3.64, 2.58 | 43.6% | 0.170 | Serious ^a^ | Not serious | Not serious | Very serious ^c, d^ | Not serious | **⨁◯◯◯**  **Very low** |
|  | GGT | Adults | Betaine/ Control | 3 | 117(61/56) | Random/MD | -3.05 | -6.85, 0.75 | 5.4% | 0.347 | Serious ^a^ | Not serious | Not serious | Very serious ^c, d^ | Not serious | **⨁◯◯◯**  **Very low** |
| Zhu,2022 | ALT | Metabolic disorders | Anthocyanins/Placebo | 12 | 893(NR) | Random/WMD | -0.92 | -4.19, 2.35 | 91.3% | <0.001 | Serious ^a^ | Very serious ^b^ | Not serious | Serious ^d^ | Not serious | **⨁◯◯◯**  **Very low** |
|  | AST | Metabolic disorders | Anthocyanins/Placebo | 12 | 893(NR) | Random/WMD | -1.22 | -3.43, 0.99 | 87.0% | <0.001 | Serious ^a^ | Very serious ^b^ | Not serious | Serious ^d^ | Not serious | **⨁◯◯◯**  **Very low** |
| Tao,2019 | ALT | Antituberculosis Drug-Induced Liver Injury | Silymarin/Placebo | 3 | 747(NR) | Fixed/SMD | − 0.15 | −0.24, −0.07 | 0% | 0.817 | Serious ^a^ | Not serious | Not serious | Not serious | Not serious | **⨁⨁⨁◯**  **Moderate** |
|  | AST | Antituberculosis Drug-Induced Liver Injury | Silymarin/Placebo | 3 | 747(NR) | Fixed/SMD | -0.14 | -0.23, -0.06 | 0% | 0.583 | Serious ^a^ | Not serious | Not serious | Not serious | Not serious | **⨁⨁⨁◯**  **Moderate** |
|  | ALP | Antituberculosis Drug-Induced Liver Injury | Silymarin/Placebo | 3 | 747(NR) | Fixed/SMD | −0.12 | −0.20, −0.03 | 39.7% | 0.127 | Serious ^a^ | Not serious | Not serious | Not serious | Not serious | **⨁⨁⨁◯**  **Moderate** |
| Wei,2013 | ALT | chronic hepatitis B | Silymarin/ Protection liver drugs | 2 | 330(200/130) | Fixed/MD | −6.11 | −12.83, 0.61 | 0% | 0.37 | Serious ^a^ | Not serious | Not serious | Serious ^d^ | Not mention | **⨁⨁◯◯**  **Low** |
|  | AST | chronic hepatitis B | silymarin /Protection liver drugs | 2 | 210(140/70) | Fixed/MD | -3.18 | -15.03,8.67 | 77% | 0.04 | Serious ^a^ | Very serious ^b^ | Not serious | Serious ^d^ | Not mention | **⨁◯◯◯**  **Very low** |
|  | ALT | Chronic hepatitis B | silymarin plus protection liver drugs/Protection liver drugs | 3 | 231(117/114) | Fixed/MD | -28.04 | -34.00, -22.09 | 46% | < 0.00001 | Serious ^a^ | Not serious | Not serious | Serious | Not mention | **⨁⨁◯◯**  **Low** |
|  | AST | Chronic hepatitis B | Silymarin plus protection liver drugs/Protection liver drugs | 3 | 231(117/114) | Fixed/MD | -34.51 | -38.77, -30.25 | 0% |  | Serious ^a^ | Not serious | Not serious | Serious | Not mention | **⨁⨁◯◯**  **Low** |
| Yang, 2014 | ALT | Chronic hepatitis C virus infection patients | Silymarin/Placebo | 2 | 212(126/76) | Fixed/MD | -6.15 | -22.03, 9.73 | 0% | 0.70 | Serious ^a^ | Not serious | Not serious | Serious ^d^ | Not mention | **⨁⨁◯◯**  **Low** |
| [Kalopitas](https://pubmed.ncbi.nlm.nih.gov/?term=Kalopitas+G&cauthor_id=33418491),2021 | ALT | NAFLD | Silymarin/Placebo | 7 | 518(262/256) | Random/MD | -14.86 | -19.37, -10.36 | 39% | <0.00001 | Serious ^a^ | Not serious | Not serious | Not serious | Not mention | **⨁⨁⨁◯**  **Moderate** |
|  | AST | NAFLD | Silymarin/Placebo | 7 | 518(262/256) | Random/MD | -7.11 | -14.16, -0.05 | 88% | <0.05 | Serious ^a^ | Very serious ^b^ | Not serious | Not serious | Not mention | **⨁◯◯◯**  **Very low** |
| Haghighat,2022 | AST | Adults | Conjugated linoleic acid/Placebo, control diet | 19 | 848(443/441) | Random/WMD | 0.41 | -0.89, 1.71 | 59.2% | 0.001 | Serious ^a^ | Serious ^e^ | Not serious | Serious ^d^ | Not serious | **⨁◯◯◯**  **Very low** |
|  | ALT | Adults | Conjugated linoleic acid/Placebo, control diet | 19 | 848(443/441) | Random/WMD | 0.59 | -0.72, 1.92 | 37.6% | 0.050 | Serious ^a^ | Not serious | Not serious | Serious ^d^ | Not serious | **⨁⨁◯◯**  **Low** |
| Soltani,2023 | ALP | Adults | Resveratrol/Placebo | 15 | 926(NR) | Random/WMD | 2.56 | -0.52, 5.64 | 39.5% | 0.06 | Serious ^a^ | Not serious | Not serious | Serious ^d^ | Not serious | **⨁⨁◯◯**  **Low** |
|  | ALT | Adults | Resveratrol/Placebo | 33 | 1747(NR) | Random/WMD | -0.91 | -2.58, 0.77; | 86.8% | <0.001 | Serious ^a^ | Very serious ^b^ | Not serious | Serious ^d^ | Not serious | **⨁◯◯◯**  **Very low** |
|  | AST | Adults | Resveratrol/Placebo | 31 | 1607(NR) | Random/WMD | -2.05 | -4.13, 0.03 | 93.2% | <0.001 | Serious ^a^ | Very serious ^b^ | Not serious | Serious ^d^ | Not serious | **⨁◯◯◯**  **Very low** |
|  | GGT | Adults | Resveratrol/Placebo | 18 | NR | Random/WMD | -0.90 | -2.68, 0.87 | 77.3% | <0.001 | Serious ^a^ | Very serious ^b^ | Not serious | Serious ^d^ | Not serious | **⨁◯◯◯**  **Very low** |
| [**Rafiee**](https://pubmed.ncbi.nlm.nih.gov/?term=Rafiee+S&cauthor_id=33321448)**,2021** | ALT | NAFLD | Resveratrol/Placebo | 5 | 216(108/108) | Random/SMD | -0.26 | -0.58,0.06 | 26% | 0.25 | Serious ^a^ | Not serious | Not serious | Serious ^d^ | Not serious | **⨁⨁◯◯**  **Low** |
|  | AST | NAFLD | Resveratrol/Placebo | 5 | 216(108/108) | Random/SMD | -0.38 | -0.84, 0.08 | 62% | 0.03 | Serious ^a^ | Serious ^e^ | Not serious | Serious ^d^ | Not serious | **⨁◯◯◯**  **Very low** |
|  | GGT | NAFLD | Resveratrol/Placebo | 3 | 136(68/68) | Random/SMD | -0.24 | -0.60, 0.13 | 12% | 0.32 | Serious ^a^ | Not serious | Not serious | Serious ^d^ | Not serious | **⨁⨁◯◯**  **Low** |
|  | ALP | NAFLD | Resveratrol/Placebo | 2 | 76(38/38) | Random/SMD | 0.24 | -0.21,0.70 | 0% | 0.46 | Serious ^a^ | Not serious | Not serious | Serious ^d^ | Not serious | **⨁⨁◯◯**  **Low** |
| Li, 2022 | AST | Individuals with any health condition | Tocotrienols/Placebo | 4 | 196(112/84) | Random/SMD | -0.195 | -0.460, 0.071 | 1.0% | 0.387 | Serious ^a^ | Not serious | Not serious | Very serious ^c, d^ | Not mention | **⨁◯◯◯**  **Very low** |
|  | ALT | Individuals with any health condition | Tocotrienols/Placebo | 5 | 436(232/204) | Random/SMD | -0.183 | -0.534, 0.169 | 66.3% | 0.018 | Serious ^a^ | Serious ^e^ | Not serious | Serious ^d^ | Not mention | **⨁◯◯◯**  **Very low** |
| Vadarlis, 2021 | AST | NAFLD | Vitamin E/Placebo | 7 | 465(232/233) | Random/MD | -5.71 | -9.49, -1.93 | 68% | 0.003 | Serious ^a^ | Very serious ^b^ | Not serious | Not serious | Not mention | **⨁◯◯◯**  **Very low** |
|  | ALT | NAFLD | Vitamin E/Placebo | 7 | 465(232/233) | Random/MD | -7.37 | -10.11, -4.64 | 0% | <0.000001 | Serious ^a^ | Not serious | Not serious | Not serious | Not mention | **⨁⨁⨁◯**  **Moderate** |
| Rezaei,2021 | ALT | NAFLD | Vitamin D/Placebo | 16 | NR | Random/WMD | −4.03 | −7.41, −0.66 | 78.5% | <0.000 | Serious ^a^ | Very serious ^b^ | Not serious | Not serious | Not serious | **⨁◯◯◯**  **Very low** |
|  | AST | NAFLD | Vitamin D/Placebo | 15 | NR | Random/WMD | -1.05 | −3.50, 1.39 | 78.2% | <0.000 | Serious ^a^ | Very serious ^b^ | Not serious | Not serious | Not serious | **⨁◯◯◯**  **Very low** |
|  | ALP | NAFLD | Vitamin D/Placebo | 7 |  | Random/WMD | -5.43% | −18.08, 7.22 | 62.4% | 0.014 | Serious ^a^ | Serious ^e^ | Not serious | Serious ^d^ | Not serious | **⨁◯◯◯**  **Very low** |
|  | GGT | NAFLD | Vitamin D/Placebo | 4 |  | Random/WMD | -2.24 | −9.66, 5.18 | 0% | 0.827 | Serious ^a^ | Not serious | Not serious | Serious ^d^ | Serious ^f^ | **⨁◯◯◯**  **Very low** |
| Asbaghi, 2021 | AST | T2DM | Chromium/Placebo | 3 | 94(59/35) | Random/WMD | -0.42 | −1.39, 0.55 | 0% | 0.630 | Serious ^a^ | Not serious | Not serious | Very serious ^c, d^ | Not serious | **⨁◯◯◯**  **Very low** |
|  | ALT | T2DM | Chromium/Placebo | 4 | 160(NR) | Random/WMD | 2.11 | − 0.69, 4.91 | 74.3% | 0.009 | Serious ^a^ | Not serious | Not serious | Very serious ^c, d^ | Serious ^f^ | **⨁◯◯◯**  **Very low** |
| Pirmadah, 2020 | ALT | Adults | L-carnitine/Placebo, no treatment | 16 | 1046 | Random/MD | −8.65 | −13.40, -3.90 | 96.2% | < 0.001 | Serious ^a^ | Very serious ^b^ | Not serious | Not serious | Not serious | **⨁◯◯◯**  **Very low** |
|  | AST | Adults | L-carnitine/Placebo, no treatment | 17 | 1042 | Random/MD | −8.52 | −12.16, -4.89 | 93.8% | <0.001 | Serious ^a^ | Very serious ^b^ | Not serious | Not serious | Serious ^f^ | **⨁◯◯◯**  **Very low** |
|  | GGT | Adults | L-carnitine/Placebo, no treatment | 5 | NR | Random/MD | −8.80 | −13.67, -3.92 | 37.8% | 0.169 | Serious ^a^ | Not serious | Not serious | Not serious | Not serious | **⨁⨁⨁◯**  **Moderate** |
| Oh, 2022 | AST | Chronic Liver Disease | L-Carnitine/ Placebo, no L-Carnitine | 10 | 710(360/350) | Random/MD | −15.84 | −24.56, −7.13 | 94% | 0.004 | Serious ^a^ | Very serious ^b^ | Not serious | Not serious | Not serious | **⨁◯◯◯**  **Very low** |
|  | ALT | Chronic Liver Disease | L-Carnitine/ Placebo, no L-Carnitine | 10 | 710(360/350) | Random/MD | −11.99 | −22.48, −1.49 | 96% | 0.03 | Serious ^a^ | Very serious ^b^ | Not serious | Not serious | Not serious | **⨁◯◯◯**  **Very low** |
| Zhang,2022 | ALT | Adults | High-protein diet/ Standard protein diet, conventional diet | 5 | 558(NR) | Random/SMD | -0.37 | -0.75, 0.01 | 76.5% | 0.002 | Serious ^a^ | Very serious ^b^ | Not serious | Serious ^d^ | Not serious | **⨁◯◯◯**  **Very low** |
|  | AST | Adults | High-protein diet/ Standard protein diet, conventional diet | 5 | 558(NR) | Random/SMD | -0.23 | -0.42, -0.03 | 15.2% | 0.318 | Serious ^a^ | Not serious | Not serious | Not serious | Not serious | **⨁⨁⨁◯**  **Moderate** |
| Ahn,2019 | AST | NAFLD | low carbohydrate diet/Low fat diet group | 4 | 130(67/63) | Random/SMD | 0.61 | -0.18,1.40 | 77% | 0.005 | Serious ^a^ | Very serious ^b^ | Not serious | Serious ^d^ | Not mention | **⨁◯◯◯**  **Very low** |
|  | ALT | NAFLD | low carbohydrate diet/Low fat diet group | 5 | 165(85/80) | Random/SMD | 0.50 | -0.13,1.14 | 73% | 0.005 | Serious ^a^ | Serious ^e^ | Not serious | Serious ^d^ | Not mention | **⨁◯◯◯**  **Very low** |
| Sangouni,2022 | AST | Adults | Mediterranean diet /Low-fat diet, energy restriction diet, American Diabetes Association diet and without any dietary treatment | 4 | 168(NR) | Random/WMD | −0.38 | − 0.73, −0.03 | 36.01% | 0.19 | Serious ^a^ | Not serious | Not serious | Serious ^c^ | Not serious | **⨁⨁◯◯**  **Low** |
|  | GGT | Adults | Mediterranean diet /Low-fat diet, energy restriction diet, American Diabetes Association diet and without any dietary treatment | 8 | 484(NR) | Random/WMD | -0.16 | -0.32, -0.006 | 0.00 | 0.76 | Serious ^a^ | Not serious | Not serious | Not serious | Not serious | **⨁⨁⨁◯**  **Moderate** |
|  | ALT | Adults | Mediterranean diet /Low-fat diet, energy restriction diet, American Diabetes Association diet and without any dietary treatment | 9 | 465(NR) | Random/WMD | -0.55 | -1.25,0.13 | 93.20% | <0.001 | Serious ^a^ | Very serious ^b^ | Not serious | Serious ^d^ | Not serious | **⨁◯◯◯**  **Very low** |
| Haigh,2022 | ALT | NAFLD | Mediterranean diet /Standard care | 9 | 418(225/193) | Random/MD | -6.54 | -12.02, -1.05 | 81% | 0.02 | Serious ^a^ | Very serious ^b^ | Not serious | Not serious | Not mention | **⨁◯◯◯**  **Very low** |
|  | ALT | NAFLD | Mediterranean components diet /Standard care | 3 | 210(104/106) | Random/MD | -5.99 | -12.93,0.95 | 74% | 0.09 | Serious ^a^ | Serious ^e^ | Not serious | Very serious ^c, d^ | Not mention | **⨁◯◯◯**  **Very low** |
|  | ALT | NAFLD | Calorie restriction diet /Standard care | 8 | 667(349/318) | Random/MD | -5.44 | -8.01, -2.88 | 0% | <0.0001 | Serious ^a^ | Not serious | Not serious | Not serious | Not mention | **⨁⨁⨁◯**  **Moderate** |
|  | AST | NAFLD | Mediterranean diet /Standard care | 6 | 314(168/146) | Random/MD | -3.40 | -7.18, 0.37 | 81% | 0.08 | Serious ^a^ | Very serious ^b^ | Not serious | Serious ^d^ | Not mention | **⨁◯◯◯**  **Very low** |
|  | AST | NAFLD | Mediterranean components diet /Standard care | 3 | 210(104/106) | Random/MD | -3.21 | -7.84,1.41 | 85% | 0.17 | Serious ^a^ | Very serious ^b^ | Not serious | Very serious ^c, d^ | Not mention | **⨁◯◯◯**  **Very low** |
|  | AST | NAFLD | Calorie restriction diet /Standard care | 7 | 628(319/309) | Random/MD | -1.61 | -3.82,0.60 | 53% | 0.15 | Serious ^a^ | Serious ^e^ | Not serious | Serious ^d^ | Not mention | **⨁◯◯◯**  **Very low** |
| Xiong,2021 | AST | NAFLD | Soy diet/Control | 3 | 172(88/84) | Random/SMD | 0.01 | -0.47, 0.49 | 57% | 0.97 | Serious ^a^ | Serious ^e^ | Not serious | Very serious ^c, d^ | Not mention | **⨁◯◯◯**  **Very low** |
|  | ALT | NAFLD | Soy diet/Control | 3 | 172(88/84) | Random/SMD | -0.01 | -0.61,0.60 | 73% | 0.98 | Serious ^a^ | Serious ^e^ | Not serious | Very serious ^c, d^ | Not mention | **⨁◯◯◯**  **Very low** |
| Chen,2019 | AST | liver cirrhosis | Late evening snacks/ Non-late evening snacks | 5 | 218(104/114) | Random/SMD | -0.284 | -0.553, -0.004 | 0% | 0.948 | Serious ^a^ | Not serious | Not serious | Serious ^c^ | Not serious | **⨁⨁◯◯**  **Low** |
|  | ALT | liver cirrhosis | Late evening snacks/ Non-late evening snacks | 3 | 113(54/59) | Random/SMD | -0.425 | -0.796, -0.055 | 0% | 0.599 | Serious ^a^ | Not serious | Not serious | Serious ^c^ | Not serious | **⨁⨁◯◯**  **Low** |
| Hallajzadeh, 2021 | AST | NR | Propolis/Placebo | 5 | 313(163/150) | Random/WMD | -5.63 | −10.59, −0.67 | 87.9% | <0.000 | Serious ^a^ | Very serious ^b^ | Not serious | Not serious | Not mention | **⨁◯◯◯**  **Very low** |
|  | ALT | NR | Propolis/Placebo | 5 | 313(163/150) | Random/WMD | -3.09 | −5.15, −1.03 | 77.1% | 0.002 | Serious ^a^ | Very serious ^b^ | Not serious | Not serious | Not mention | **⨁◯◯◯**  **Very low** |
| Mostafa Qorbani, 2022 | AST | Adults | Almond/ Placebo, no intervention | 4 |  | Random/SMD | -0.20 | -0.43, 0.04 | 00.7% | 0.388 | Serious ^a^ | Not serious | Not serious | Serious ^d^ | Not mention | **⨁⨁◯◯**  **Low** |
|  | ALT | Adults | Almond/ Placebo, no intervention | 5 |  | Random/SMD | -0.16 | -0.43, 0.10 | 43.4% | 0.133 | Serious ^a^ | Not serious | Not serious | Serious ^d^ | Not mention | **⨁⨁◯◯**  **Low** |
|  | GGT | Adults | Almond/ Placebo, no intervention | 3 |  | Random/SMD | 0.02 | -0.28, 0.32 | 29.4% | 0.243 | Serious ^a^ | Not serious | Not serious | Serious ^d^ | Not mention | **⨁⨁◯◯**  **Low** |
| Lu,2016 | ALT | NAFLD | Omega-3 Fatty Acid/Placebo, no treatment | 8 | 530(282/248) | Random/MD | -4.97 | -11.14,1.20 | 59% | 0.11 | Serious ^a^ | Serious ^e^ | Not serious | Not serious | Serious ^f^ | **⨁◯◯◯**  **Very low** |
|  | AST | NAFLD | Omega-3 Fatty Acid/Placebo, no treatment | 7 | 496(265/231) | Random/MD | -2.01 | -8.72,4.70 | 84% | 0.56 | Serious ^a^ | Very serious ^b^ | Not serious | Not serious | Serious ^f^ | **⨁◯◯◯**  **Very low** |
|  | GGT | NAFLD | Omega-3 Fatty Acid/Placebo, no treatment | 4 | 237(132/105) | Random/MD | -9.02 | -14.80, -3.24 | 0% | 0.002 | Serious ^a^ | Not serious | Not serious | Serious ^c^ | Serious ^f^ | **⨁⨁◯◯**  **Low** |
| Golzan，2023 | AST | Adults | Non-nutritive sweetener/Placebo | 10 | 813 | Random/WMD | 0.02 | –1.26, 1.30 | 74.6% | < 0.001 | Serious ^a^ | Serious ^e^ | Not serious | Serious ^d^ | Not serious | **⨁◯◯◯**  **Very low** |
|  | ALT | Adults | Non-nutritive sweetener/Placebo | 10 | 793 | Random/WMD | –0.78, | –2.14, 0.57 | 80.5% | < 0.001 | Serious ^a^ | Very serious ^b^ | Not serious | Serious ^d^ | Not serious | **⨁◯◯◯**  **Very low** |
|  | GGT | Adults | Non-nutritive sweetener/Placebo | 5 | 433 | Random/WMD | –0.21 | –1.46, 1.04 | 0.00% | 0.485 | Serious ^a^ | Not serious | Not serious | Serious ^d^ | Not serious | **⨁⨁◯◯**  **Low** |

Abbreviations: MD: mean difference；SMD standard mean difference；RR: relative risk; OR: odd risk; NR：not reported; I: intervention; C: comparator; NAFLD, non-alcoholic fatty liver diseases; T2DM, type 2 diabetes mellitus;

◯ represented “low”; ⨁ represented “high”.

^a^ The included study had an unclear risk of selection, performance, detection, and reporting biases; ^b^ I^2^ ≥ 75%; ^c^ Sample size <300; ^d^ 95 % Cl includes invalid line; ^e^ 50% ≤ I^2^ < 75%; ^f^ Funnel plot or Egger's or Begg's tests indicated asymmetry.

References:

Ahn, J., Jun, D. W., Lee, H. Y., & Moon, J. H. 2019. Critical appraisal for low-carbohydrate diet in nonalcoholic fatty liver disease: Review and meta-analyses. *Clinical Nutrition (Edinburgh, Scotland),* 38(5), 2023-30. doi:10.1016/j.clnu.2018.09.022

Asbaghi, O., Ghanbari, N., Shekari, M., Reiner, Ž., Amirani, E., Hallajzadeh, J., . . . Asemi, Z. 2020. The effect of berberine supplementation on obesity parameters, inflammation and liver function enzymes: A systematic review and meta-analysis of randomized controlled trials. *Clinical Nutrition ESPEN,* 38, 43-49. doi:10.1016/j.clnesp.2020.04.010

Asbaghi, O., Kashkooli, S., Mardani, M., Rezaei Kelishadi, M., Fry, H., Kazemi, M., & Kaviani, M. 2021. Effect of green coffee bean extract supplementation on liver function and inflammatory biomarkers: A meta-analysis of randomized clinical trials. *Complementary Therapies In Clinical Practice,* 43, 101349. doi:10.1016/j.ctcp.2021.101349

Asbaghi, O., Naeini, F., Ashtary-Larky, D., Kaviani, M., Rezaei Kelishadi, M., Eslampour, E., . . . Naeini, A. A. 2021. Effects of chromium supplementation on blood pressure, body mass index, liver function enzymes and malondialdehyde in patients with type 2 diabetes: A systematic review and dose-response meta-analysis of randomized controlled trials. *Complementary Therapies In Medicine,* 60, 102755. doi:10.1016/j.ctim.2021.102755

Ashtary-Larky, D., Bagheri, R., Ghanavati, M., Asbaghi, O., Tinsley, G. M., Mombaini, D., . . . Wong, A. 2022. Effects of betaine supplementation on cardiovascular markers: A systematic review and Meta-analysis. *Critical Reviews In Food Science and Nutrition,* 62(23), 6516-33. doi:10.1080/10408398.2021.1902938

Chen, C.-J., Wang, L.-C., Kuo, H.-T., Fang, Y.-C., & Lee, H.-F. 2019. Significant effects of late evening snack on liver functions in patients with liver cirrhosis: A meta-analysis of randomized controlled trials. *Journal of Gastroenterology and Hepatology,* 34(7), 1143-52. doi:10.1111/jgh.14665

Ghaffar, S., Naqvi, M. A., Fayyaz, A., Abid, M. K., Khayitov, K. N., Jalil, A. T., . . . Nouri, M. 2022. What is the influence of grape products on liver enzymes? A systematic review and meta-analysis of randomized controlled trials. *Complementary Therapies In Medicine,* 69, 102845. doi:10.1016/j.ctim.2022.102845

Ghafouri, A., Estêvão, M. D., Alibakhshi, P., Pizarro, A. B., Kashani, A. F., Persad, E., . . . Morvaridzadeh, M. 2021. Sumac fruit supplementation improve glycemic parameters in patients with metabolic syndrome and related disorders: A systematic review and meta-analysis. *Phytomedicine : International Journal of Phytotherapy and Phytopharmacology,* 90, 153661. doi:10.1016/j.phymed.2021.153661

Ghavami, A., Ziaei, R., Foshati, S., Hojati Kermani, M. A., Zare, M., & Amani, R. 2020. Benefits and harms of ginseng supplementation on liver function? A systematic review and meta-analysis. *Complementary Therapies In Clinical Practice,* 39, 101173. doi:10.1016/j.ctcp.2020.101173

Golzan, S. A., Movahedian, M., Haghighat, N., Asbaghi, O., & Hekmatdoost, A. 2023. Association between non-nutritive sweetener consumption and liver enzyme levels in adults: a systematic review and meta-analysis of randomized clinical trials. *Nutrition Reviews*. doi:10.1093/nutrit/nuac107

Haghighat, N., Shimi, G., Shiraseb, F., Karbasi, A., Nadery, M., Ashtary-Larky, D., . . . Asbaghi, O. 2022. The effects of conjugated linoleic acid supplementation on liver function enzymes and malondialdehyde in adults: A GRADE-assessed systematic review and dose-response meta-analysis. *Pharmacological Research,* 186, 106518. doi:10.1016/j.phrs.2022.106518

Haigh, L., Kirk, C., El Gendy, K., Gallacher, J., Errington, L., Mathers, J. C., & Anstee, Q. M. 2022. The effectiveness and acceptability of Mediterranean diet and calorie restriction in non-alcoholic fatty liver disease (NAFLD): A systematic review and meta-analysis. *Clinical Nutrition (Edinburgh, Scotland),* 41(9), 1913-31. doi:10.1016/j.clnu.2022.06.037

Hallajzadeh, J., Milajerdi, A., Amirani, E., Attari, V. E., Maghsoudi, H., & Mirhashemi, S. M. 2021. Effects of propolis supplementation on glycemic status, lipid profiles, inflammation and oxidative stress, liver enzymes, and body weight: a systematic review and meta-analysis of randomized controlled clinical trials. *Journal of Diabetes and Metabolic Disorders,* 20(1), 831-43. doi:10.1007/s40200-020-00696-w

Kalopitas, G., Antza, C., Doundoulakis, I., Siargkas, A., Kouroumalis, E., Germanidis, G., . . . Chourdakis, M. 2021. Impact of Silymarin in individuals with nonalcoholic fatty liver disease: A systematic review and meta-analysis. *Nutrition (Burbank, Los Angeles County, Calif.),* 83, 111092. doi:10.1016/j.nut.2020.111092

Kamel, A. M., & Farag, M. A. 2022. Therapeutic Potential of Artichoke in the Treatment of Fatty Liver: A Systematic Review and Meta-Analysis. *Journal of Medicinal Food,* 25(10), 931-42. doi:10.1089/jmf.2022.0025

Karimi, E., Farrokhzad, A., Darand, M., & Arab, A. 2021. The Effect of Saffron Consumption on Liver Function: A Systematic Review and Meta-Analysis of Randomized Controlled Clinical Trials. *Complementary Medicine Research,* 28(5), 453-62. doi:10.1159/000515003

Li, F., Xu, B., Soltanieh, S., Zanghelini, F., Abu-Zaid, A., & Sun, J. 2022. The effects of tocotrienols intake on obesity, blood pressure, inflammation, liver and glucose biomarkers: a meta-analysis of randomized controlled trials. *Critical Reviews In Food Science and Nutrition,* 62(26), 7154-67. doi:10.1080/10408398.2021.1911926

Lu, W., Li, S., Li, J., Wang, J., Zhang, R., Zhou, Y., . . . Guo, C. 2016. Effects of Omega-3 Fatty Acid in Nonalcoholic Fatty Liver Disease: A Meta-Analysis. *Gastroenterology Research and Practice,* 2016, 1459790. doi:10.1155/2016/1459790

Mahmoodi, M., Hosseini, R., Kazemi, A., Ofori-Asenso, R., Mazidi, M., & Mazloomi, S. M. 2020. Effects of green tea or green tea catechin on liver enzymes in healthy individuals and people with nonalcoholic fatty liver disease: A systematic review and meta-analysis of randomized clinical trials. *Phytotherapy Research : PTR,* 34(7), 1587-98. doi:10.1002/ptr.6637

Morvaridzadeh, M., Qorbani, M., Shokati Eshkiki, Z., Estêvão, M. D., Mohammadi Ganjaroudi, N., Toupchian, O., . . . Ziaei, S. 2022. The effect of almond intake on cardiometabolic risk factors, inflammatory markers, and liver enzymes: A systematic review and meta-analysis. *Phytotherapy Research : PTR,* 36(12), 4325-44. doi:10.1002/ptr.7622

Mousavi, S. M., Jayedi, A., Bagheri, A., Zargarzadeh, N., Wong, A., Persad, E., . . . Koohdani, F. 2021. What is the influence of cinnamon supplementation on liver enzymes? A systematic review and meta-analysis of randomized controlled trials. *Phytotherapy Research : PTR,* 35(10), 5634-46. doi:10.1002/ptr.7200

Ngu, M. H., Norhayati, M. N., Rosnani, Z., & Zulkifli, M. M. 2022. Curcumin as adjuvant treatment in patients with non-alcoholic fatty liver (NAFLD) disease: A systematic review and meta-analysis. *Complementary Therapies In Medicine,* 68, 102843. doi:10.1016/j.ctim.2022.102843

Oh, H., Park, C. H., & Jun, D. W. 2022. Impact of l-Carnitine Supplementation on Liver Enzyme Normalization in Patients with Chronic Liver Disease: A Meta-Analysis of Randomized Trials. *Journal of Personalized Medicine,* 12(7). doi:10.3390/jpm12071053

Panjeshahin, A., Mollahosseini, M., Panbehkar-Jouybari, M., Kaviani, M., Mirzavandi, F., & Hosseinzadeh, M. 2020. Effects of garlic supplementation on liver enzymes: A systematic review and meta-analysis of randomized controlled trials. *Phytotherapy Research : PTR,* 34(8), 1947-55. doi:10.1002/ptr.6659

Pirmadah, F., Ramezani-Jolfaie, N., Mohammadi, M., Talenezhad, N., Clark, C. C. T., & Salehi-Abargouei, A. 2020. Does L-carnitine supplementation affect serum levels of enzymes mainly produced by liver? A systematic review and meta-analysis of randomized controlled clinical trials. *European Journal of Nutrition,* 59(5), 1767-83. doi:10.1007/s00394-019-02068-4

Rafiee, S., Mohammadi, H., Ghavami, A., Sadeghi, E., Safari, Z., & Askari, G. 2021. Efficacy of resveratrol supplementation in patients with nonalcoholic fatty liver disease: A systematic review and meta-analysis of clinical trials. *Complementary Therapies In Clinical Practice,* 42, 101281. doi:10.1016/j.ctcp.2020.101281

Rastkar, M., Nikniaz, L., Abbasalizad Farhangi, M., & Nikniaz, Z. 2022. Systematic review and meta-analysis of the effect of garlic in patients with non-alcoholic fatty liver disease. *Indian Journal of Gastroenterology : Official Journal of the Indian Society of Gastroenterology,* 41(6), 548-57. doi:10.1007/s12664-022-01287-8

Razmpoosh, E., Safi, S., Abdollahi, N., Nadjarzadeh, A., Nazari, M., Fallahzadeh, H., . . . Salehi-Abargouei, A. 2020. The effect of Nigella sativa on the measures of liver and kidney parameters: A systematic review and meta-analysis of randomized-controlled trials. *Pharmacological Research,* 156, 104767. doi:10.1016/j.phrs.2020.104767

Rezaei, S., Tabrizi, R., Nowrouzi-Sohrabi, P., Jalali, M., Shabani-Borujeni, M., Modaresi, S., . . . Doaei, S. 2021. The Effects of Vitamin D Supplementation on Anthropometric and Biochemical Indices in Patients With Non-alcoholic Fatty Liver Disease: A Systematic Review and Meta-analysis. *Frontiers In Pharmacology,* 12, 732496. doi:10.3389/fphar.2021.732496

Sangouni, A. A., Hassani Zadeh, S., Mozaffari-Khosravi, H., & Hosseinzadeh, M. 2022. Effect of Mediterranean diet on liver enzymes: a systematic review and meta-analysis of randomised controlled trials. *The British Journal of Nutrition,* 128(7), 1231-39. doi:10.1017/S0007114521002270

Soltani, S., Sharifi-Zahabi, E., Sangsefidi, Z. S., Ahmadi Vasmehjani, A., Meshkini, F., Clayton, Z. S., & Abdollahi, S. 2023. The effect of resveratrol supplementation on biomarkers of liver health: A systematic review and meta-analysis of randomized controlled trials. *Phytotherapy Research : PTR,* 37(3), 1153-66. doi:10.1002/ptr.7719

Tang, G., Zhang, L., Tao, J., & Wei, Z. 2021. Effect of Nigella sativa in the treatment of nonalcoholic fatty liver disease: A systematic review and meta-analysis of randomized controlled trials. *Phytotherapy Research : PTR,* 35(8), 4183-93. doi:10.1002/ptr.7080

Tao, L., Qu, X., Zhang, Y., Song, Y., & Zhang, S.-X. 2019. Prophylactic Therapy of Silymarin (Milk Thistle) on Antituberculosis Drug-Induced Liver Injury: A Meta-Analysis of Randomized Controlled Trials. *Canadian Journal of Gastroenterology & Hepatology,* 2019, 3192351. doi:10.1155/2019/3192351

Vadarlis, A., Antza, C., Bakaloudi, D. R., Doundoulakis, I., Kalopitas, G., Samara, M., . . . Chourdakis, M. 2021. Systematic review with meta-analysis: The effect of vitamin E supplementation in adult patients with non-alcoholic fatty liver disease. *Journal of Gastroenterology and Hepatology,* 36(2), 311-19. doi:10.1111/jgh.15221

Wei, F., Liu, S. K., Liu, X. Y., Li, Z. J., Li, B., Zhou, Y. L., . . . Li, Y. W. 2013. Meta-analysis: silymarin and its combination therapy for the treatment of chronic hepatitis B. *European Journal of Clinical Microbiology & Infectious Diseases : Official Publication of the European Society of Clinical Microbiology,* 32(5), 657-69. doi:10.1007/s10096-012-1789-1

Wei, X., Wang, C., Hao, S., Song, H., & Yang, L. 2016. The Therapeutic Effect of Berberine in the Treatment of Nonalcoholic Fatty Liver Disease: A Meta-Analysis. *Evidence-based Complementary and Alternative Medicine : ECAM,* 2016, 3593951. doi:10.1155/2016/3593951

Xiong, P., & Zhu, Y.-F. 2021. Soy diet for nonalcoholic fatty liver disease: A meta-analysis of randomized controlled trials. *Medicine,* 100(22), e25817. doi:10.1097/MD.0000000000025817

Yang, Z., Zhuang, L., Lu, Y., Xu, Q., & Chen, X. 2014. Effects and tolerance of silymarin (milk thistle) in chronic hepatitis C virus infection patients: a meta-analysis of randomized controlled trials. *BioMed Research International,* 2014, 941085. doi:10.1155/2014/941085

Zhang, Y., Chen, X., Allison, D. B., & Xun, P. 2022. Efficacy and safety of a specific commercial high-protein meal-replacement product line in weight management: meta-analysis of randomized controlled trials. *Critical Reviews In Food Science and Nutrition,* 62(3), 798-809. doi:10.1080/10408398.2020.1829539

Zhou, F., She, W., He, L., Zhu, J., & Gu, L. 2022. The effect of anthocyanins supplementation on liver enzymes among patients with metabolic disorders: A systematic review and meta-analysis of randomized clinical trials. *Phytotherapy Research : PTR,* 36(1), 53-61. doi:10.1002/ptr.7280
